# Fast ion transport for synthesis and stabilization of *β*-Zn_4_Sb_3_

**DOI:** 10.1038/s41467-021-26265-0

**Published:** 2021-10-19

**Authors:** Dongwang Yang, Xianli Su, Jian He, Yonggao Yan, Jun Li, Hui Bai, Tingting Luo, Yamei Liu, Hao Luo, Yimeng Yu, Jinsong Wu, Qingjie Zhang, Ctirad Uher, Xinfeng Tang

**Affiliations:** 1 State Key Laboratory of Advanced Technology for Materials Synthesis and Processing, Wuhan University of Technology, 430070 Wuhan, China; 2 Department of Physics and Astronomy, Clemson University, Clemson, SC 29634 USA; 3 Nanostructure Research Centre, Wuhan University of Technology, 430070 Wuhan, China; 4 Department of Physics, University of Michigan, Ann Arbor, MI 48109 USA

**Keywords:** Materials chemistry, Thermoelectric devices and materials, Thermoelectrics

## Abstract

Mobile ion-enabled phenomena make *β*-Zn_4_Sb_3_ a promising material in terms of the re-entry phase instability behavior, mixed electronic ionic conduction, and thermoelectric performance. Here, we utilize the fast Zn^2+^ migration under a sawtooth waveform electric field and a dynamical growth of 3-dimensional ionic conduction network to achieve ultra-fast synthesis of *β*-Zn_4_Sb_3_. Moreover, the interplay between the mobile ions, electric field, and temperature field gives rise to exquisite core-shell crystalline-amorphous microstructures that self-adaptively stabilize *β*-Zn_4_Sb_3_. Doping Cd or Ge on the Zn site as steric hindrance further stabilizes *β*-Zn_4_Sb_3_ by restricting long-range Zn^2+^ migration and extends the operation temperature range of high thermoelectric performance. These results provide insight into the development of mixed-conduction thermoelectric materials, batteries, and other functional materials.

## Abstract (web summary)

*β*-Zn_4_Sb_3_ has promising thermoelectric performance, but its ionic migration properties make it prone to degradation. Here the authors exploit the ion migration in an electric field-assisted synthesis method, fast producing *β*-Zn_4_Sb_3_ with improved phase stability and extended temperature range for the thermoelectric operation.

## Introduction

Mass transport, along with energy and charge transfer, is ubiquitous in nature and underscores diverse phenomena in science, engineering, and technology. Ionic migration is an important form of mass transport. On the one hand, electric field-driven ionic migration enables a wide range of applications such as batteries, fuel cells, and sensors^1^. On the other hand, ionic migration risks phase instability, for example, precipitation occurs when the mobile ion’s electrochemical potential is higher than its atomic counterpart. For the sake of phase stability, the voltage across an ionic conductor needs to be lower than the material-specific temperature-dependent threshold^2^.

Here arise two profound questions. First, whether the mass transport in the form of ionic migration can be utilized towards fast materials synthesis. Mass transport mechanisms, different from the traditional thermal diffusion, are a frontier topic of materials science and engineering^3^. Second, whether the interplay between mobile ions, thermal, and electric fields can yield certain microstructures that stabilize the phase at a level beyond the aforementioned “threshold”.

*β*-Zn_4_Sb_3_ is a perfect material template to address these questions. *β*-Zn_4_Sb_3_ is known for its complex crystal structure as well as the coexistence of mixed electronic and ionic conduction and promising thermoelectric (TE) performance in the low to intermediate temperature range (400–700 K)^4–13^.

Unlike most state-of-the-art TE materials that are electron-based semiconductors or semimetals^14,15^, the mobile ions in *β*-Zn_4_Sb_3_ play a vital role in its promising TE performance in the context of the Electron-Crystal Phonon-Liquid paradigm^16^. The weakly bonded Zn interstitials enable highly mobile Zn^2+^ and also ultra-low thermal conductivity^17–20^. However, broader commercial applications of *β*-Zn_4_Sb_3_ face two long-standing obstacles: developing time- and cost-efficient synthesis recipes and maintaining good phase stability^21–28^ while delivering outstanding TE performance.

To overcome these two *β*-Zn_4_Sb_3_-specific obstacles and to answer the two aforementioned general questions, here we have utilized the fast migration of Zn^2+^ ions under electrical field and a dynamical growth of three-dimensional (3D) ionic conduction network to attain ultra-fast synthesis of *β*-Zn_4_Sb_3_. The presence of an electric field and the dynamic formation of 3D ionic conduction network speeded up the mass diffusion process and the chemical reaction of Zn and Sb into *β*-Zn_4_Sb_3_ compared to the typical temperature-driven diffusion and chemical reactions. Due to the rapid cooling of the ion channel bulge when the current is cut off, the amorphous nonstoichiometric Zn_4_Sb_3_ layer is formed and encapsulates crystalline *β*-Zn_4_Sb_3_ grain. Such a core-shell crystalline-amorphous micromorphology, especially the amorphous grain boundary, restricts the migration of Zn^2+^ ions across grains, and thus suppressed Zn precipitates. Hence, the as-formed core-shell microstructures self-adaptively suppress the thermodynamic re-entry instability of *β*-Zn_4_Sb_3_. Finally, doping by Cd or Ge on the Zn site as a steric hindrance further inhibits the migration of Zn^2+^ ions within each grain. All these mechanisms worked jointly to improve the phase stability of *β*-Zn_4_Sb_3_ and extend the operating temperature range of high TE performance.

## Results and discussion

### Fast synthesis

**Fig. 1:**
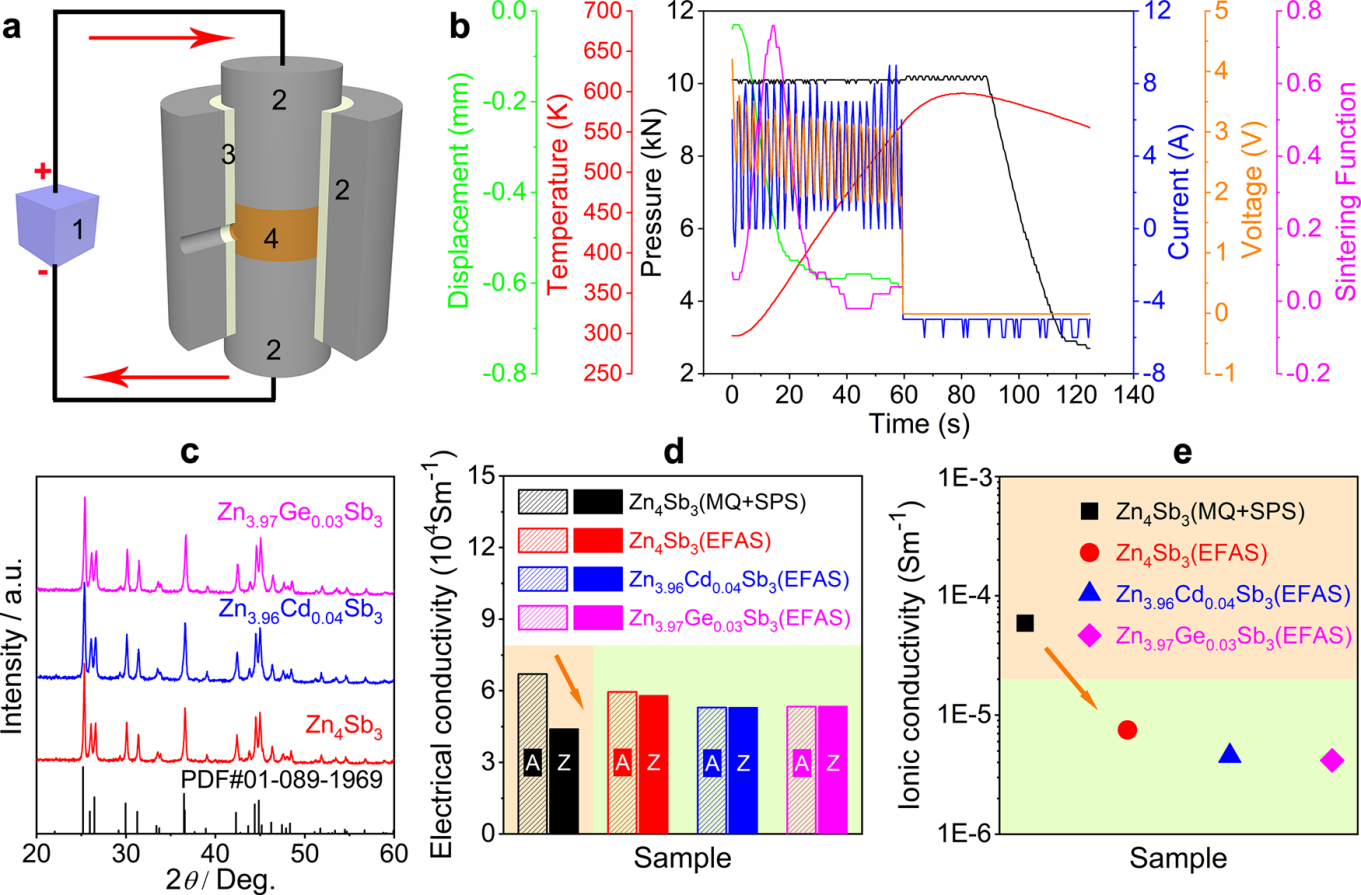
Ultra-fast synthesis, composition, and properties of *β*-Zn4Sb3-based materials. **a** Schematic diagram of the reaction apparatus, “1–4” represents the DC pulse power supply, graphite die, BN layer, stoichiometric admixture of Zn and Sb powders, respectively. **b** Time profile of the reaction parameters, including the loading pressure, temperature of the admixture, displacement of the graphite punch, current, voltage, and sintering function (i.e., the derivative of displacement). **c** Phase composition of the synthesized samples (standard XRD spectra of PDF#01-089-1969). **d** Room temperature electrical conductivity of MQ + SPS Zn_4_Sb_3_, EFAS Zn_4_Sb_3_, EFAS Zn_3.96_Cd_0.04_Sb_3_, and EFAS Zn_3.97_Ge_0.03_Sb_3_ samples before and after a test cycle; “A” and “Z” represent the sample at the beginning and end of the electrical conductivity test. **e** Room temperature Zn^2+^ ionic conductivities of MQ + SPS Zn_4_Sb_3_, EFAS Zn_4_Sb_3_, EFAS Zn_3.96_Cd_0.04_Sb_3_, and EFAS Zn_3.97_Ge_0.03_Sb_3_ samples (orange arrow indicating the decline of ionic conductivity).

Single-phase pristine *β*-Zn_4_Sb_3_ was formed quickly through an electric field-assisted synthesis (EFAS) process. A schematic diagram of the EFAS apparatus is shown in Fig. 1a. The stoichiometric admixture of Zn and Sb powders was loaded into a graphite die, the inner wall of which was coated with a layer of BN to ensure that maximum current passed through the admixture rather than the die wall. A thermocouple was inserted into the admixture to measure the reaction temperature. The system is evacuated and maintained at a vacuum level ≤20 Pa. The (Zn_1 − *x*_Cd_*x*_)_4_Sb_3_ (*x* = 0.005, 0.01, and 0.015) and (Zn_1 − *x*_Ge_*x*_)_4_Sb_3_ (*x* = 0.0025, 0.005, and 0.0075) samples were synthesized under the same EFAS condition as pristine *β*-Zn_4_Sb_3_.

Figure 1b displays the key reaction parameters, including the loading pressure, the temperature of the admixture, displacement of the graphite punch, current, voltage, and sintering function (i.e., the derivative of the displacement), as a function of time. The amplitude of current and voltage with a sawtooth waveform and a frequency of 0.4 HZ were applied in the range of 0–9 A and 1.8–3.6 V, respectively. The highest temperature measured during the passage of the current was 598 K, much lower than the growth-from-the-melt temperature of 1023 K^4,5^, and the SPS synthesis temperature of ~700 K^29,30^ for *β*-Zn_4_Sb_3_ based materials. At about 10 s after the current was on, the sintering function peaked, suggesting the onset of reaction. At ~20 s, the displacement of the graphite punch reached its maximum. After 60 s, Zn_4_Sb_3_, Zn_3.96_Cd_0.04_Sb_3_, and Zn_3.97_Ge_0.03_Sb_3_ compounds were obtained with a relative density higher than 98%, as shown in Fig. 1c and Supplementary Figs. 1 and 2.

A reference pristine sample of *β*-Zn_4_Sb_3_ was prepared using the melt-quench method followed by spark plasma sintering (hereafter named the MQ + SPS sample). The MQ + SPS sample was prepared from high purity elemental Zn shots (5N) and Sb chunks (5N), mixed and sealed in a silica tube under a pressure of 10^−3^ Pa. The tube was slowly heated up to 1023 K and dwelt for 12 h, followed by quenching in saltwater. The obtained ingot was hand-ground into fine powders and consolidated using an SPS apparatus at 713 K for 4 min under a pressure of 30 MPa. The relative packing density of the as-prepared pellets, with a diameter of 16 mm and a thickness of 3 mm, was 97.6%. Supplementary Fig. 3 shows the phase composition of Zn_4_Sb_3_ (MQ + SPS). The X-ray diffraction pattern confirmed the single-phased nature of *β*-Zn_4_Sb_3_ (rhombohedral structure).

### Robust properties

*β*-Zn_4_Sb_3_ is a high-performance TE material made of cheap and relatively nontoxic elements. TE materials directly convert heat into electricity through the Seebeck effect, or function as heat pumps via the Peltier effect, thereby playing an important role in our global package of renewable energy options^14,15^. The efficiency of a TE material is gauged by its dimensionless figure of merit *ZT*, defined as *ZT* = *α*^2^*σT*/(*κ*_L_ + *κ*_e_), where *α*, *σ*, *κ*_L_, *κ*_e_, and *T* are the Seebeck coefficient, electrical conductivity, lattice thermal conductivity, electronic thermal conductivity, and the absolute temperature, respectively^14,15^. The detailed TE transport measurements of the Zn_4_Sb_3_-based samples were performed, as shown in Supplementary Figs. 4–12. The EFAS Zn_4_Sb_3_-based compounds show excellent TE performance. For the EFAS Zn_3.96_Cd_0.04_Sb_3_ sample, the maximum *ZT* value of 1.2 is achieved at 693 K (cf. Supplementary Fig. 7), while for the EFAS Zn_3.97_Ge_0.03_Sb_3_ sample, the maximum *ZT* value of 1.12 is achieved at 700 K (cf. Supplementary Fig. 10). Upon doping with Cd or Ge, the overall *ZT* value increases in comparison with that of the pristine Zn_4_Sb_3_ sample in the entire range of temperatures (cf. Supplementary Figs. 7 and 10).

In history, the promising TE performance of *β*-Zn_4_Sb_3_-based compounds is overshadowed by the phase instability between 425 and 565 K^18^, and also the severe ion migration and Zn precipitates (kinetics instability) under an applied electric field even at room temperature (cf. Supplementary Fig. 13). For example, the MQ + SPS Zn_4_Sb_3_ sample shows very different TE properties when measured twice, and the sample undergoes irreversible change during the measurement (cf. Supplementary Fig. 14).

In contrast, the electrical conductivity, Seebeck coefficient, thermal conductivity, and calculated *ZT* curves of the EFAS Zn_4_Sb_3_-based compounds are very repeatable and reproducible (cf. Supplementary Figs. 15–20). The room temperature electrical conductivity of all the samples before and after a test cycle is presented in Fig. 1d. It is obvious that the electrical conductivity of the MQ + SPS Zn_4_Sb_3_ sample decreased significantly from 6.7 × 10^4^ to 4.4 × 10^4^ Sm^−1^, and that of EFAS Zn_4_Sb_3_ sample decreased slightly from 5.9 × 10^4^ to 5.8 × 10^4^ Sm^−1^, while that of EFAS Zn_3.96_Cd_0.04_Sb_3_ sample and that of EFAS Zn_3.97_Ge_0.03_Sb_3_ sample remain almost unchanged at about 5.3 × 10^4^ to 5.4 × 10^4^ Sm^−1^, respectively.

We also measured the Zn^2+^ conductivity and ran endurance tests under high current density at elevated temperatures. We employed a direct-current (DC) polarization method with electron-blocking electrodes to isolate the ionic conduction by filtering out the electronic conduction^2,31,32^. Commercial zinc-loaded montmorillonite served as the electrodes. As shown in Supplementary Fig. 21, the Zn^2+^ migration rate is found to be 3.3 × 10^−3^ Sm^−1^ at room temperature by the alternating-current (AC) electrochemical impedance spectroscopy (EIS)^33–36^. Solid-state Au|zinc-loaded montmorillonite|Zn_4_Sb_3_-based compounds|zinc-loaded montmorillonite|Au pseudo-galvanic cell was constructed (cf. Supplementary Fig. 22), in which all Zn_4_Sb_3_-based samples have a similar size of 8 × 8 × 1.3 mm^3^. When the output voltage is close to 10 V, which is the device limit, the current is reduced from 25 μA in the MQ + SPS Zn_4_Sb_3_, to 3.5 μA in the EFAS Zn_4_Sb_3_, to 1.9 μA in the EFAS Zn_3.96_Cd_0.04_Sb_3_, and finally to <1.9 μA in the EFAS Zn_3.97_Ge_0.03_Sb_3_, as shown in Supplementary Figs. 23–26. The room temperature ionic conductivities of MQ + SPS Zn_4_Sb_3_, EFAS Zn_4_Sb_3_, EFAS Zn_3.96_Cd_0.04_Sb_3_, and EFAS Zn_3.97_Ge_0.03_Sb_3_ samples were calculated to be 5.9 × 10^−5^, 7.5 × 10^−6^, 4.5 × 10^−6^, and 4.2 × 10^−6^ Sm^−1^, respectively (cf. Fig. 1e). Hence, the Zn^2+^ conductivity in the EFAS samples is suppressed by an order of magnitude compared to the MQ + SPS sample, which partially explains the observed improvement in the phase stability and the reproducibility of transport data.

Doping Cd or Ge further enhanced the phase stability as evidenced by the results of electromigration tests conducted at a temperature of 473 K, a DC current density of 20 A/cm^2^, and a charging time of 24 h (cf. Supplementary Figs. 27 and 28). For comparison, the electromigration test was also carried out on the MQ + SPS sample under the same conditions. Supplementary Figs. 29–32 show the fracture surface morphology of typical samples after the electromigration tests. Obvious cracking and precipitation of Zn whiskers are observed on the MQ + SPS Zn_4_Sb_3_ sample (cf. Supplementary Fig. 29). In contrast, the grain surface of the EFAS Zn_4_Sb_3_ sample only becomes rough with no discernible precipitation of Zn (cf. Supplementary Fig. 30), and the grain surface of the EFAS Zn_3.96_Cd_0.04_Sb_3_ sample is very clean with only a small number of microcracks (cf. Supplementary Fig. 31). Even better results are obtained with the EFAS Zn_3.97_Ge_0.03_Sb_3_ sample, which shows no cracks but a clean surface (cf. Supplementary Fig. 32).

The above results corroborate that the chemical stability of the EFAS samples is greatly improved. Combined with doping at the Zn sites, particularly doping with Ge, makes the EFAS samples exceptionally stable even under a high DC electric field. In the following sections, we will pinpoint the microscopic origins of the phase formation and the phase stability.

### Phase formation mechanism

To pinpoint the phase formation mechanism of *β*-Zn_4_Sb_3_ in the EFAS process, we first need to differentiate the effect of heating (nondirectional) and the effect of electric field (directional). Specially, we pay close attention to two specific aspects:

**Aspect #1** Temporal window of phase formation.

Single phased *β*-Zn_4_Sb_3_ can be obtained within 60 s under a pulsed DC field. However, when the charging time extends past 83 s (cf. Supplementary Fig. 33), it is observed that ZnSb forms at the upstream side of the sample, and Zn whiskers precipitate at the sample’s downstream side. We invert the die and the sample (turned upside down) and charge for 18 s (cf. Supplementary Fig. 34), the Zn and ZnSb are gone, and a homogeneous *β*-Zn_4_Sb_3_ sample re-emerges. These observations corroborate that (i) the as-formed *β*-Zn_4_Sb_3_ serves as a fast transport channel of Zn^2+^ ions, and (ii) there is a temporal window for the phase formation of *β*-Zn_4_Sb_3_, beyond which the decomposition occurs in the presence of an electric current.

**Aspect #2** The process of phase formation.

**Fig. 2:**
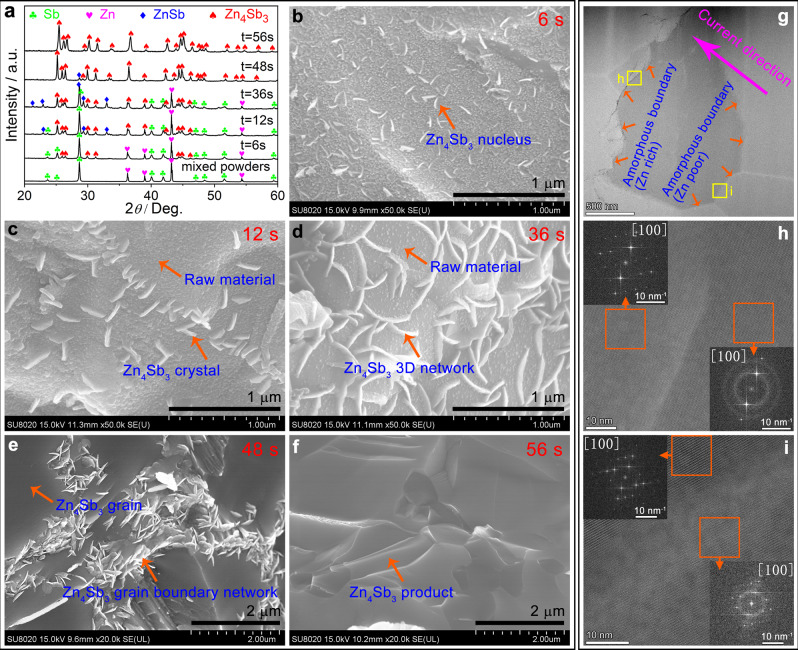
Phase transformation and microstructure evolution during the ultra-rapid synthesis of *β*-Zn_4_Sb_3_ bulk material. **a** Temporal evolution of the *β*-Zn_4_Sb_3_ phase formation; microstructure transformation process of *β*-Zn_4_Sb_3_ by FESEM: **b** 6 s, **c** 12 s, **d** 36 s, **e** 48 s, **f** 56 s. Fine microstructure of *β*-Zn_4_Sb_3_ by TEM: **g** macroscopic image of grain, **h** macroscopic image of grain boundary located at the downstream side of the current, and the illustration is the diffraction pattern in the orange frame area, **i** macroscopic image of grain boundary located at the upstream side of current, and the illustration is the diffraction pattern in the orange frame area.

The phase formation of *β*-Zn_4_Sb_3_ is the result of the competition between field-assisted phase formation and phase decomposition. Figure 2a depicts a number of intermediate stages in the process of phase formation of *β*-Zn_4_Sb_3_. More detailed experimental results are provided in Supplementary Fig. 41. When Zn and Sb powders are in physical contact, a small amount of *β*-Zn_4_Sb_3_ is formed under the pulsed electric field within less than 10 s, the process is controlled by mutual inter-diffusion of Zn and Sb. Between 10 and 36 s, both the amount of ZnSb and *β*-Zn_4_Sb_3_ increase simultaneously, as reflected by the peak in the sintering function curve in Fig. 1b. The appearance of ZnSb suggests that the transport speed of Zn^2+^ ions is so fast in the ion channels of *β*-Zn_4_Sb_3_ that Zn cannot be replenished in a timely manner so some are as-formed *β*-Zn_4_Sb_3_ decomposes back into ZnSb and Zn. Then, the amount of ZnSb gradually decreases while that of *β*-Zn_4_Sb_3_ further increases, which reflects the fact that the as-formed *β*-Zn_4_Sb_3_ forms a 3D network and makes more Zn available to react with ZnSb into *β*-Zn_4_Sb_3_. Finally, *β*-Zn_4_Sb_3_ is attained at the time of 56 s.

The microstructural evolution confirms that the as-formed *β*-Zn_4_Sb_3_ serves as a fast transport channel of Zn^2+^ ions, first form nuclei, and crystals, and then form a 3D network, through which Zn^2+^ can be transported quickly under the electric field (cf. Figure 2b–f). Finer microstructure details of *β*-Zn_4_Sb_3_ were investigated by TEM (cf. Figure 2g–i). The crystal grain shows a perfect *β*-Zn_4_Sb_3_ lattice, while the grain boundary appears to be amorphous. The amorphous grain boundary located at the downstream side of current is rich in Zn (cf. Fig. 2h), while the counterpart at the upstream side of current is poor in Zn (cf. Fig.e 2i), de facto forming a special core-shell structure composite.

**Fig. 3:**
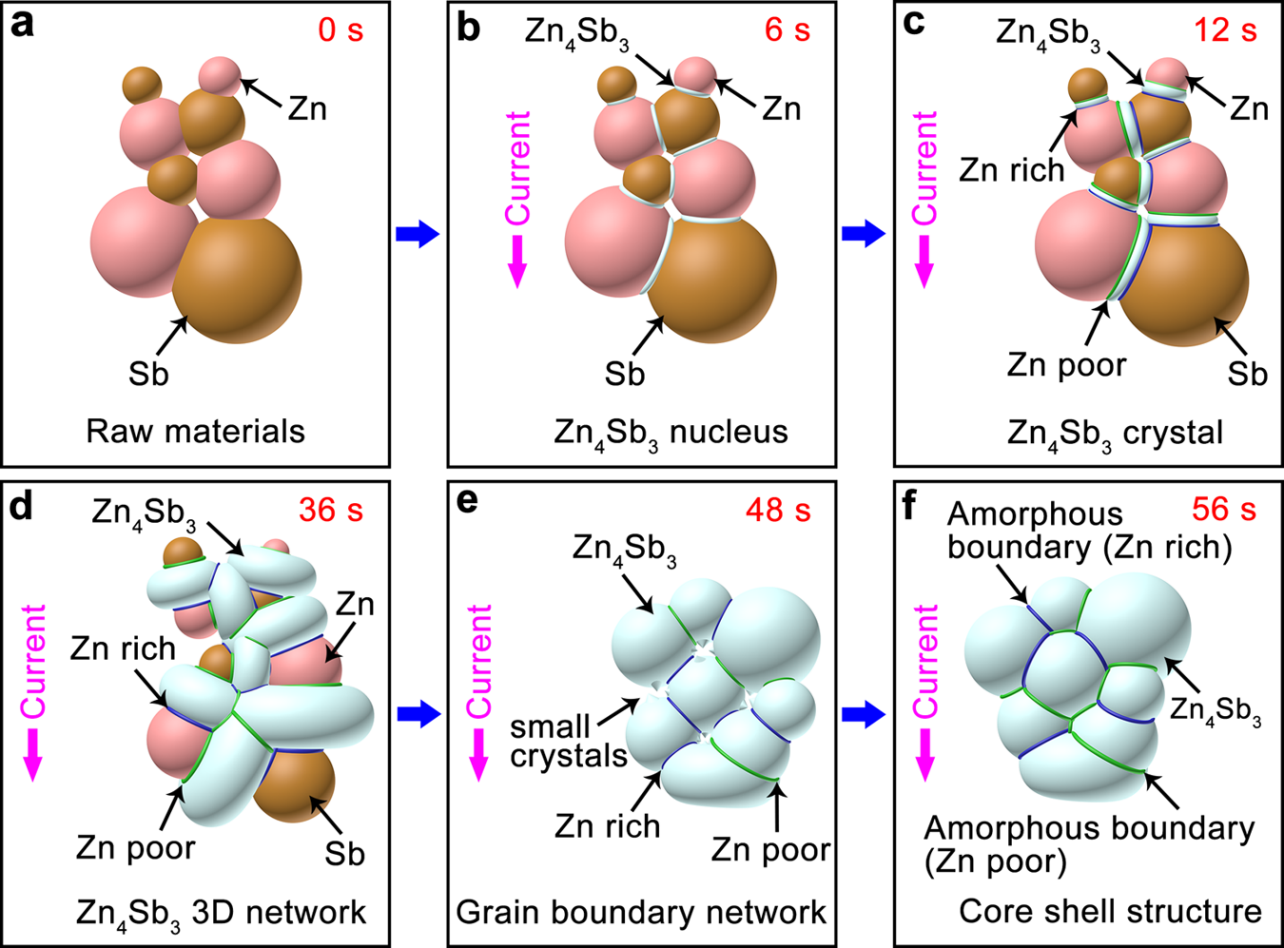
Schematic diagram of the reaction mechanism. **a** Raw materials of Zn and Sb powders with stoichiometric ratio. **b** *β*-Zn_4_Sb_3_ nuclei are formed at the contact point between Zn and Sb particles when the current is loaded (6 s). **c** *β*-Zn_4_Sb_3_ crystal grows up gradually, and the composition in the upstream region of the current is poor in Zn, while the composition in the downstream region is rich in Zn (12 s). **d** *β*-Zn_4_Sb_3_ 3D network is formed as growing *β*-Zn_4_Sb_3_ crystals contact each other (36 s). **e** Special grain boundary network structure filled with a large number of *β*-Zn_4_Sb_3_ nanocrystals is formed due to the ion channel bulge. **f** Core-shell microstructure consisted of crystalline *β*-Zn_4_Sb_3_ grains and amorphous off-stoichiometric Zn_4_Sb_3_ grain boundaries that are formed when the current is off.

These results point toward a schematic diagram of the reaction mechanism as shown in Fig. 3a–f. When Zn is in physical contact with Sb, under the pulse current, *β*-Zn_4_Sb_3_ nucleus forms (cf. Fig. 3b). Due to the high ionic conductivity of Zn^2+^ in *β*-Zn_4_Sb_3_, Zn^2+^ ions are easily transported through the as-formed *β*-Zn_4_Sb_3_ and react with Sb forming additional *β*-Zn_4_Sb_3_ in spite of Zn being separated from Sb by *β*-Zn_4_Sb_3_ (cf. Fig. 3c). Due to the transport speed of Zn^2+^ in the ion channel of *β*-Zn_4_Sb_3_ mismatches the local supply of fresh Zn, the upstream and downstream side of grain boundaries tend to be, respectively, poor and rich in Zn along the current direction. As *β*-Zn_4_Sb_3_ crystals contact each other (at ~36 s), a 3D network is formed (cf. Figure 3d), which greatly promotes the transport speed and scope of Zn^2+^ ions and makes *β*-Zn_4_Sb_3_ grains grow rapidly. In particular, a large number of *β*-Zn_4_Sb_3_ nanocrystals are formed at the ion channel bulges, these nanocrystals fill the voids between Zn_4_Sb_3_ grains, and lead to the densification of material (cf. Fig. 3e). Normally, the current discharge at the grain boundary produces highly intensive Joule heating. When the current is off, the rapid cooling at the contact quenches nanocrystals to amorphous, forming the core-shell crystalline-amorphous microstructure (cf. Fig. 3f).

### Phase stability mechanism

The core-shell crystalline-amorphous micromorphology somewhat explains the observed improvement of chemical stability of the EFAS samples because the amorphous layer is not ionic conducting. More details are provided by the in situ transmission electron microscopy study. The responses to an electric field applied to MQ + SPS Zn_4_Sb_3_ sample and EFAS Zn_4_Sb_3_ sample are recorded in detail in the Supporting information (cf. Supplementary Movies 1 and 2).

**Fig. 4:**
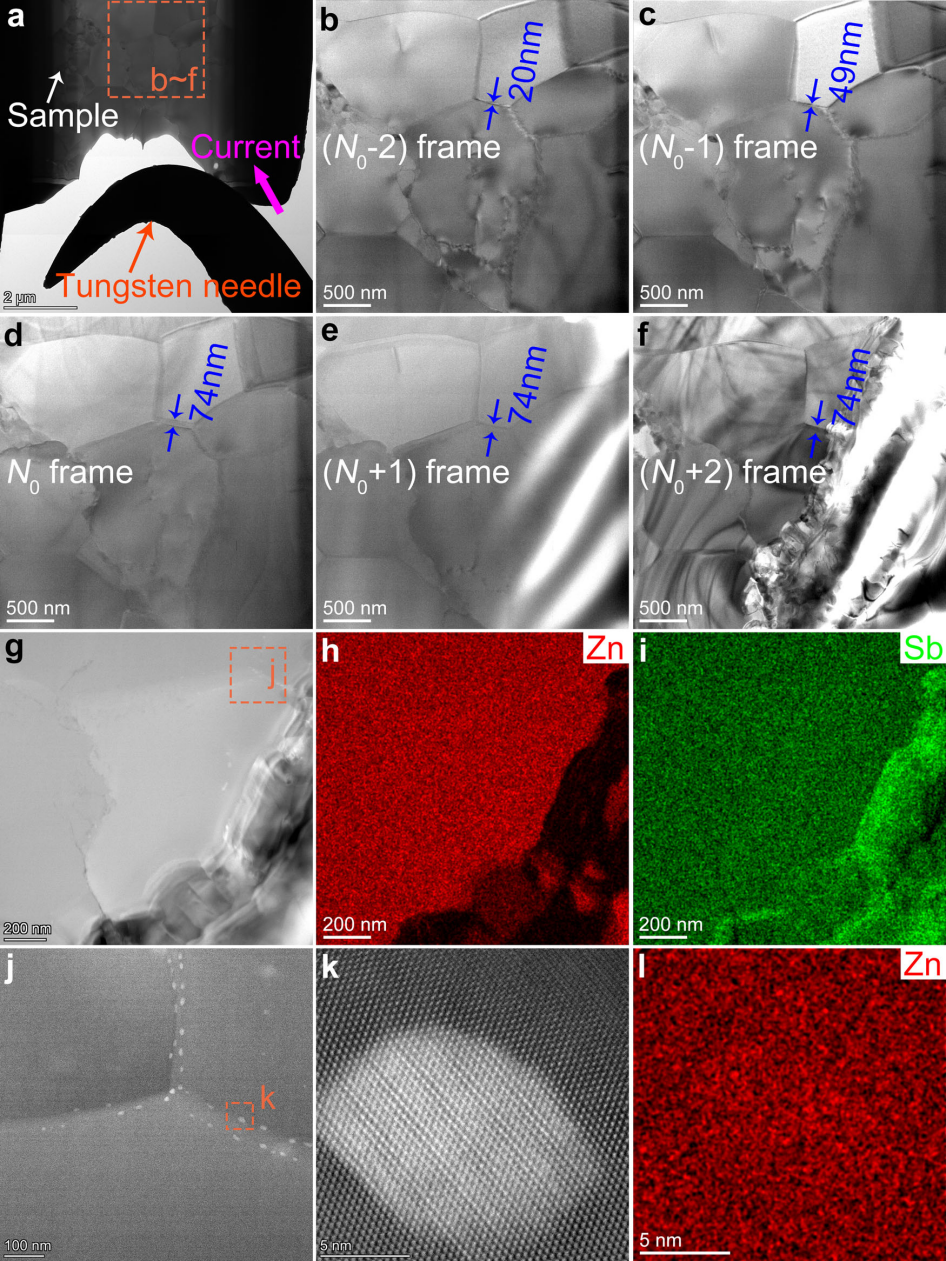
In situ microstructure evolution. **a**–**f** Different stages (in time) during the current flow through the EFAS Zn_4_Sb_3_ sample (the grain boundary reaches the widest at the *N*_0_ frame). **g**–**i** The HAADF image and the corresponding EDS spectrum of EFAS Zn_4_Sb_3_ sample after the current are turned off. **j** Enlarged HAADF image of the grain boundary area delineated by an orange border in (**g**); white particles are precipitated at the junction of the two grains. **k**–**l** High-resolution images and EDS spectra of white particles.

In addition, Fig. 4a–f shows different stages (in time) when the current flows through the EFAS Zn_4_Sb_3_ sample. When the applied voltage exceeds 0.69 V, the grain boundary is gradually widened, increasing from ~20 to ~74 nm, and, finally, the grain collapses and the material becomes unstable. Figure 4g–i shows the high-angle annular dark-field (HAADF) image and its corresponding energy-dispersive X-ray spectroscopy (EDS) spectrum of the EFAS Zn_4_Sb_3_ sample after the current is turned off. It is obvious that the Zn^2+^ ions migrate downstream along the current, and the upstream grains crack and fragment due to the loss of Zn. Fine structures of the grain boundary were studied (cf. Fig. 4j–l). White particles, aka Zn metal, are precipitated at the junction of the two grains (cf. Fig. 4l) and are amorphous (cf. Fig. 4k). Interestingly, the surrounding areas of the white precipitates become crystalline. Apparently, when the applied voltage on the material is higher than the critical voltage, the amorphous grain boundary crystallizes into *β*-Zn_4_Sb_3_, which conducts Zn^2+^ ions under the electric field and finally destabilizes the grain. On the contrary, as long as a voltage is applied, the grains of MQ + SPS Zn_4_Sb_3_ sample will crack and disintegrate continually, and there is no phenomenon of grain boundary widening (cf. Supplementary Fig. 45 and Supplementary Movie 2).

**Fig. 5:**
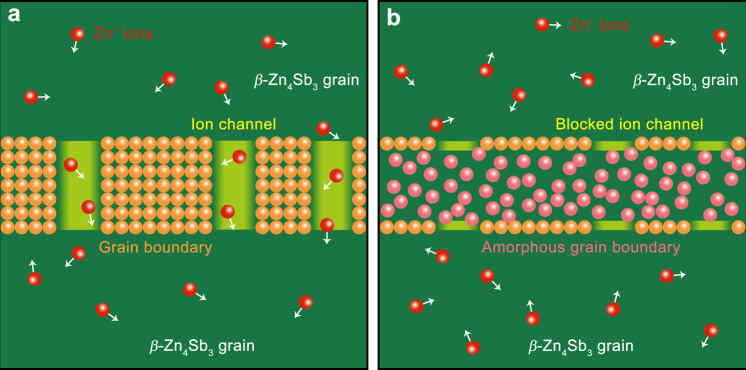
Ion blocking mechanism. **a** Ion channels run through the grains to form a network all over the MQ + SPS Zn_4_Sb_3_ bulk material. **b** Ion channels in EFAS Zn_4_Sb_3_ bulk material are limited to the interior of the grain, and the amorphous layer on the grain boundary (including rich Zn and poor Zn) blocks the formation of the network due to the lack of ion channel.

Figure 5 shows the schematic diagram of *β*-Zn_4_Sb_3_ microstructure derived by different synthesis methods. For MQ + SPS Zn_4_Sb_3_ sample, ion channels run through the grains to the entire bulk as there is no ion migration blocking mechanism (cf. Fig. 5a). While for the EFAS Zn_4_Sb_3_ sample, ion channels are limited to the interior of the grain, and the amorphous layer on the grain boundary (respectively, poor and rich in Zn) blocks the formation of the network due to the lack of ion channels, so as to improve the stability of the bulk material (cf. Fig. 5b). Moreover, the literatures^5,37,38^ show that foreign elements can replace dynamic Zn, and thus reduce the ionic conduction and thus improve the stability. To sum up, the core-shell crystalline-amorphous microstructure combined with doping by foreign elements (i.e., Cd or Ge) at the Zn sites inhibit the migration of dynamic Zn^2+^ ions at multiple scales, and improve the thermodynamic (phase transition, cf. Supplementary Figs. 43 and 44) and kinetic (ionic migration, cf. Supplementary Figs. 29–32) stability of *β*-Zn_4_Sb_3_.

In summary, we have prepared a series of *β*-Zn_4_Sb_3_-based compounds with different dopants by an EFAS technique. The mechanism of phase formation and decomposition, and chemical stability, along with the TE transport properties, have been discussed in detail. Under the DC electric field, the ion transport channel in *β*-Zn_4_Sb_3_ enables fast mass transport and participates in chemical reactions. A dense EFAS *β*-Zn_4_Sb_3_-based bulk material can be formed within 60 s. The resulting bulk composite material has a crystal-amorphous core-shell micromorphology. The as-formed composite microstructure, in conjunction with Cd/Ge doping, stabilizes the high TE performance of *β*-Zn_4_Sb_3_ via suppressing the long-range Zn ions migration and precipitates of Zn on the grain boundaries. Specifically, the Zn^2+^ ionic conductivity is decreased by more than an order magnitude from 5.9 × 10^−5^ Sm^−1^ for the MQ + SPS sample to 4.2 × 10^−6^ Sm^−1^ for the Ge-doped structure. Moreover, *β*-Zn_4_Sb was doped with 0.75 at.% Ge shows essentially no change when carrying the current density of 20 A/cm^2^ at 473 K for 24 h. The Cd-doped structure Zn_3.96_Cd_0.04_Sb_3_ attained *ZT*_max_ = 1.2, at 693 K, while the Ge-doped Zn_3.97_Ge_0.03_Sb_3_ sample reached *ZT*_max_ = 1.12, at 700 K. The synthesis method can be in principle extended to, for example, ZnSb, Cu_2_Se, and Cu_2_S (cf. Supplementary Figs. 46–48), which is of great technical implications.

## Methods

### Synthesis

Four grams of Zn (5N, 200 mesh) and Sb (5N, 200 mesh) or Cd (5N, 200 mesh) and Ge (5N, 200 mesh) powders were weighed according to the stoichiometric ratio and were thoroughly mixed in an agate mortar for 20 min. The mixed raw powders were transferred into the mold (Φ16 mm) in Fig. 1a. The inner wall of the graphite mold was coated by a layer of BN to ensure most current flows through the powder admixture. A thermocouple was inserted into the admixture to measure the reaction temperature. In a typical process, a vacuum level ≤ 20 Pa was maintained. Next, a pulsed sawtooth-shaped current is applied to the mold for 60 s. The resulting pellet with a diameter of 16 mm and a height of 3 mm had a relative density of more than 98%. The ingot was cut into appropriate shapes for the TE property measurements and electromigration tests.

Moreover, the EFAS processes for Bi_2_Te_3_, ZnSb, Cu_2_Se, and Cu_2_S compounds are really similar.

### Test of ionic conductivity of Zn^2+^

The migration rate of Zn^2+^ ions in the commercial zinc-loaded montmorillonite was tested through AC EIS. A solid-state Au|zinc-loaded montmorillonite|Zn_4_Sb_3_-based compounds|zinc-loaded montmorillonite|Au pseudo-galvanic cell was constructed, in which all Zn_4_Sb_3_-based samples have a similar size of 8 × 8 × 1.3 mm^3^. Then, based on the DC polarization measurements, the resulting ionic conductivities of Zn_4_Sb_3_ (MQ + SPS), Zn_4_Sb_3_ (EFAS), Zn_3.96_Cd_0.04_Sb_3_ (EFAS), and Zn_3.97_Ge_0.03_Sb_3_ (EFAS) at room temperature could be obtained.

### Chemical electromigration tests

Electromigration tests were conducted at a temperature of 473 K, with the DC current density of 20 A/cm^2^, and the charging time of 24 h. High-purity argon gas of 100 Pa filled the cavity to avoid a short circuit.

### Characterization

The phase purity of all samples was inspected by X-ray powder diffraction (Empyrean, Cu K_α_ line, PANalytical, Holland). Images of freshly fractured surfaces were taken by field emission scanning electron microscopy (SU8000, Hitachi, Japan) with EDS (XFlash6160, Bruker, Germany). Back-scattered images were taken by EPMA (JXA-8100, JEOL, Japan). A direct characterization of the samples’ atomic structures and structural evolution during the process of electrification was carried out on transmission electron microscopy (Talos F200s, FEI) and double spherical aberration-corrected transmission electron microscopy (Titan Themis G2 60-300, FEI). The samples for TEM observation were prepared by ion milling with liquid nitrogen (PIPS 695, Gatan) and focused ion beam milling (Helios Nanolab G3 UC, FEI). In situ biasing experiments were performed within the TEM column using a TEM-STM holder (Pico Femto, Zeptools). The low- and high-temperature heat flow of the Zn_4_Sb_3_-based samples, respectively, was detected by Q2000 (TA, USA).

The electrical conductivity (*σ*) and the Seebeck coefficient (*α*) were measured simultaneously using a commercial equipment (ZEM-3, Ulvac, Japan). The high-temperature Hall coefficient (*R*_H_) was measured in a magnetic performance testing system (NYMS, China), using the van der Pauw method under a reversible magnetic field of 1.5 T. The low-temperature *σ* and *R*_H_ between 10 and 300 K were measured on a Physical Properties Measurement System (PPMS-9, Quantum Design, USA). The effective carrier concentration (*n*_H_) was calculated by the formula: *n*_H_ = 1/*eR*_H_, where *e* is the electron charge. The Hall mobility follows from *μ*_H_ = *σR*_H_. The thermal conductivity was calculated from the relation *κ* = *D* × *c*_p_ × *ρ*, where *D* is the thermal diffusivity coefficient, *c*_p_ is the specific heat capacity, and *ρ* is the bulk density. *D* was measured using an LFA457 (Netzsch, Germany) laser flash apparatus. *c*_p_ was taken as the Dulong–Petit law value. *ρ* was obtained by the Archimedes method.

## Supplementary information

Supplementary Information

Description of Additional Supplementary Files

Supplementary Movie 1

Supplementary Movie 2

## Data Availability

The authors declare that all data supporting the findings of this work are available from the corresponding authors upon reasonable request.
