# Supplementary material for: Fast ion transport for synthesis and stabilization of β-Zn4Sb3
Source: Nat Commun. 2021 Oct 19;12:6077. doi: 10.1038/s41467-021-26265-0 (PMC8526605; doi:10.1038/s41467-021-26265-0)
Supplement: Supplementary file 1 — Supplementary Information [file 41467_2021_26265_MOESM1_ESM.pdf]

Supplementary Information for

Fast Ion Transport for Synthesis and Stabilization of β-Zn_4Sb_3

Dongwang Yang[1], Xianli Su[1], Jian He[2*], Yonggao Yan[1], Jun Li[1], Hui Bai[1,3], Tingting Luo[1,3], Yamei Liu[2], Hao Luo[1,3], Yimeng Yu[1,3], Jinsong Wu[1,3*], Qingjie Zhang[1], Ctirad Uher[4], and Xinfeng Tang[1*]

[1]State Key Laboratory of Advanced Technology for Materials Synthesis and Processing, Wuhan University of Technology, Wuhan 430070, China.
[2]Department of Physics and Astronomy, Clemson University, Clemson, South Carolina 29634, USA.
[3]Nanostructure Research Centre, Wuhan University of Technology, Wuhan 430070, China.
[4]Department of Physics, University of Michigan, Ann Arbor, Michigan 48109, USA.

Correspondence and requests for materials should be addressed to Jian He (jianhe@g.clemson.edu), Jinsong Wu (wujs@whut.edu.cn), or to Xinfeng Tang (tangxf@whut.edu.cn)

Supplementary Note 1

Synthesis of pure Zn_4Sb_3, $(Zn_{1-x}Cd_x)_4Sb_3$ (x=0.005, 0.01, 0.015), and $(Zn_{1-x}Ge_x)_4Sb_3$ (x=0.0025, 0.005, 0.0075) dense bulk materials: 4 g of Zn (5N, 200 mesh) and Sb (5N, 200 mesh), or Cd (5N, 200 mesh), Ge (5N, 200 mesh) powders were weighed according to the stoichiometric ratio, and thoroughly mixed in an agate mortar for 20 min. The mixed raw powders were transferred into the mold (Φ16mm) in **Fig. 1a**. The inner wall of the graphite mold was coated by a layer of BN to ensure most current flows through the powder admixture. A thermocouple was inserted into the admixture to measure the reaction temperature. In a typical process, a vacuum level ≤ 20 Pa was maintained. Next, a pulsed sawtooth-shaped current is applied to the mold for 60 s. The resulting pellet with the diameter of 16 mm and the height of 3 mm had a relative density of more than 98%. The ingot was cut into appropriate shapes for the thermoelectric property measurements and electromigration tests.

Moreover, the electric field-assisted synthesis (EFAS) processes for Bi_2Te_3, ZnSb, Cu_2Se, and Cu_2S compounds are really similar.

Fig. S1 – S2 show the phase composition of $(Zn_{1-x}Cd_x)_4Sb_3$ and $(Zn_{1-x}Ge_x)_4Sb_3$, respectively. The X-ray diffraction patterns are consistent with the standard cards of the Zn_4Sb_3 rhombohedral phase, indicating that they are all single phased compounds.

Supplementary Note 2

Synthesis of pure Zn_4Sb_3 (MQ + SPS) dense bulk materials: stoichiometric amounts of high purity single-element Zn shots (5N) and Sb chunks (5N) were weighed and sealed in a silica tube under a pressure of 10^{-3} Pa. Subsequently, the tube was slowly heated up to 1023 K and maintained there for 12 h, followed by quenching in salt water. Finally, the obtained ingot was ground into fine powders and consolidated by using spark plasma sintering (SPS) apparatus at 713 K for 4 min under a pressure of 30 MPa. The relative density of the resulting pellets, having the diameter of 16 mm and the height of 3 mm, was 97.6%. **Fig. S3** shows the phase compositions of Zn_4Sb_3 (MQ + SPS). The X-ray diffraction pattern is consistent with the standard cards of β-Zn_4Sb_3 rhombohedral phase, indicating that it is a single phased compound.

Supplementary Note 3

Thermoelectric properties: The electrical conductivity (σ) and the Seebeck coefficient (α) were measured simultaneously using a commercial equipment (ZEM-3, Ulvac, Japan). The high temperature Hall coefficient (R_H) was measured in a magnetic performance testing system (NYMS, China), using the van der Pauw method under a reversible magnetic field of 1.5 T. The low temperature σ and R_H between 10 K and 300 K were measured on a Physical Properties Measurement System (PPMS-9, Quantum Design, USA). The effective carrier concentration (n_H) was calculated by the formula: $n_H=1/eR_H$, where e is the electron charge. The Hall mobility follows from $\mu_H=\sigma R_H$. The thermal conductivity was calculated from the relation $\kappa=D\times c_p\times\rho$, where D is the thermal diffusivity coefficient, c_p is the specific heat capacity, and ρ is the bulk density. D was measured using a LFA457 (Netzsch, Germany) laser flash apparatus. c_p was taken as the Dulong-Petit law value. ρ was obtained by the Archimedes method.

The uncertainties were estimated to be 3% for the thermal diffusivity and the electrical conductivity, and 5% for the Seebeck coefficient, with the overall uncertainty of 11% for the thermoelectric Fig. of merit ZT.

The carrier concentration of Zn_4Sb_3 (MQ + SPS) fluctuates violently near 423 K and 560 K (**Fig. S5a**), which means that the sample is unstable in the high temperature region. In contrast, the carrier concentration curve of Zn_4Sb_3 prepared by EFAS method is much smoother as a function of temperature (**Fig. S8a**), which documents that the sample prepared by the EFAS method has a significantly improved kinetic stability. Moreover, the carrier concentrations of $Zn_{3.96}Cd_{0.04}Sb_3$ and $Zn_{3.97}Ge_{0.03}Sb_3$ samples do not change noticeably in the entire temperature range (**Fig. S8** and **S11**), which means that an appropriate level of doping further stabilizes the compounds.

An approximate relationship $\mu_H \sim T^{-1.5}$ is observed for all samples in the high temperature range, implying that acoustic phonon scattering of charge carriers plays the dominant role (**Fig. S5, S8 and S11**).

According to the literature,[1,2] alloy scattering is expected to reduce the carrier mobility, but the results obtained in this study are quite different. The carrier mobility of $Zn_{3.96}Cd_{0.04}Sb_3$ and $Zn_{3.97}Ge_{0.03}Sb_3$ samples is much higher than that of the undoped Zn_4Sb_3 samples at all temperatures. This is likely due to a partial suppression of the

disorder among Zn atoms, which improves the kinetic stability of the samples.

Furthermore, we have tested the low temperature electrical transport properties on PPMS, the data displayed in **Fig. S6, S9 and S12**. There is a sharp inflection point in the low temperature transport curve of the Zn_4Sb_3 (MQ + SPS) sample near 230 K (**Fig. S6**), while the curve of the Zn_4Sb_3 sample prepared by the EFAS method is much smoother (**Fig. S9**). The low temperature transport curve of $Zn_{3.96}Cd_{0.04}Sb_3$ is very similar to that of Zn_4Sb_3 (EFAS) (**Fig. S9**), and a small inflection point appears near 240 K. Of course, the carrier concentration is reduced after doping with Cd. The curve for the $Zn_{3.97}Ge_{0.03}Sb_3$ sample is very flat before 240 K (**Fig. S12**), in contrast to the curves of Zn_4Sb_3 and $Zn_{3.96}Cd_{0.04}Sb_3$ EFAS samples. This may be due to changes in the band structure of Zn_4Sb_3 upon doping with Ge[2]. With the increasing temperature, the power exponent p of the temperature dependence of the carrier mobility ($\mu \sim T^{-p}$) changes gradually from 0 to 0.5, and then to 1.5, which means that alloy scattering gradually transforms into acoustic phonon scattering.

Moreover, the lattice thermal conductivity κ_L of β-Zn_4Sb_3 (EFAS) is lower than that of β-Zn_4Sb_3 (MQ+SPS) over the entire temperature range, even though the latter is more dynamically unstable (**Fig. S4 and S7**). This is due to the special composite structure of the sample prepared by the EFAS method. The amorphous grain boundary and its large number of disordered regions inside the grains, as well as the dynamic process of decomposition and re-formation of the β-Zn_4Sb_3 compound under the temperature difference, would lead to strong scattering of phonons. Furthermore, the κ_L of the $Zn_{3.97}Ge_{0.03}Sb_3$ EFAS sample is lower than that of the β-Zn_4Sb_3 EFAS compound (**Fig. S10**), which is caused by additional alloy scattering. Interestingly, the lattice thermal conductivity of the $Zn_{3.96}Cd_{0.04}Sb_3$ EFAS sample is lower than that of the $Zn_{3.97}Ge_{0.03}Sb_3$ EFAS sample (**Fig. S7 and S10**). This is likely the result of a greater content of Cd impurity, as well as its much heavier mass compared to an atom of Ge, leading to a greater mass contrast and thus stronger mass and stress field fluctuation scattering of phonons.

In addition, the thermoelectric properties of the samples were tested repeatedly, and the thermoelectric properties of new samples were re-prepared by the same EFAS process. All TE properties of different samples are shown in **Fig. S14 – S20**. The results of two test cycles of TE properties of the Zn_4Sb_3 (MQ + SPS) sample are very

different (**Fig. S14**), which indicates that the sample has experienced changes after high temperature treatment. In contrast, TE properties of the samples prepared by the EFAS method are stable even after 10 cycles of testing (**Fig. S15, S17 and S19**). In the meantime, TE properties of the re-prepared new samples are also very close to each other (**Fig. S16**, **S18** and **S20**). All these results show that the reproducibility of the samples is really good.

Supplementary Note 4

Test of ionic conductivity of Zn^{2+}: The migration rate of Zn^{2+} ions in the commercial zinc-loaded montmorillonite is 3.3×10^{-3} Sm^{-1}, as tested through AC electrochemical impedance spectroscopy (EIS)[3-6] (**Fig. S21**).

A solid-state Au|zinc-loaded montmorillonite|Zn_4Sb_3 based compounds|zinc-loaded montmorillonite|Au pseudo-galvanic cell was constructed (**Fig. S22**), in which all Zn_4Sb_3-based samples have a similar size of $8 \times 8 \times 1.3$ mm^3. **Fig. S23 - S26** show potential variation curves for Zn_4Sb_3 (MQ + SPS), Zn_4Sb_3 (EFAS), $Zn_{3.96}Cd_{0.04}Sb_3$ (EFAS), $Zn_{3.97}Ge_{0.03}Sb_3$ (EFAS), respectively. Then, based on the DC polarization measurements, the resulting ionic conductivities of Zn_4Sb_3 (MQ + SPS), Zn_4Sb_3 (EFAS), $Zn_{3.96}Cd_{0.04}Sb_3$ (EFAS), $Zn_{3.97}Ge_{0.03}Sb_3$ (EFAS) at room temperature were calculated to be 5.9×10^{-5} Sm^{-1}, 7.5×10^{-6} Sm^{-1}, 4.5×10^{-6} Sm^{-1}, 4.2×10^{-6} Sm^{-1}, respectively.

Supplementary Note 5

Chemical electromigration tests: In order to further demonstrate enhancements in the composition stability of the samples in this work, electromigration tests were conducted at a temperature of 473 K, with the DC current density of 20 A/cm^2, and the charging time of 24 h. **Fig. S27** shows a schematic diagram of the chemical electromigration experiment. An external heating wire provides a high temperature environment for the test sample after charging, and the two ends of the Zn_4Sb_3-based sample are charged with the direct current. **Fig. S28** displays the home-made chemical electromigration experimental device. High purity argon gas of 100 Pa filled the cavity to avoid short circuit.

In contrast, we applied a current density of 20 A/cm^2 through a Zn_4Sb_3 (MQ+SPS) sample. Zn precipitates formed at the downstream end of the sample after

a day at ambient conditions (**Fig. S13**). Obviously, *β*-Zn_4Sb_3 is susceptible to a kinetic instability as Zn^{2+} ions diffuse rapidly under an electric field, even at room temperature.

Fig. S29 - S32 show experimental results of high temperature chemical electromigration of Zn_4Sb_3 (MQ + SPS), Zn_4Sb_3 (EFAS), $Zn_{3.96}Cd_{0.04}Sb_3$ (EFAS), $Zn_{3.97}Ge_{0.03}Sb_3$ (EFAS) bulk material, respectively.

For Zn_4Sb_3 (MQ + SPS), the surface color has changed from silver white to dark brown (**Fig. S29**). In the downstream part of the current, the sample developed cracking and precipitates of Zn appeared on the lower surface. The precipitated Zn whiskers are seen clearly in the downstream part of the current of a fractured surface, and the micron-size grain is broken into a large number of small nanosized particles. In the grains located in upstream part of the current, there are many micro- and nano-scale sized voids. Meanwhile, there are a lot of Zn nanoparticles in the voids. It is obvious that, in a high temperature environment, the Zn_4Sb_3 (MQ + SPS) sample undergoes severe Zn^{2+} migration under DC electric fields.

For the Zn_4Sb_3 sample made with EFAS method, the surface color has become yellowish after the electromigration test (**Fig. S30**). The sample at the downstream part of the current did not crack, which is quite different from that of the Zn_4Sb_3 (MQ + SPS) sample. In addition, the fracture surface morphology of this sample in the upstream and downstream part of the current are observed. The grain surfaces become rough in the downstream part of the current. At the same time, a small number of microcracks are observed in the sample. However, no large amounts of Zn whiskers, similar to that in the Zn_4Sb_3 (MQ + SPS) sample, are found. A small number of microcracks is observed at the grain boundary of the Zn_4Sb_3 sample in the upstream part of the current, and there are no large voids similar to those in the Zn_4Sb_3 (MQ + SPS) sample.

For the $Zn_{3.96}Cd_{0.04}Sb_3$ sample, its surface color has turned light yellow after the electromigration experiment, and the color is much lighter than that of the Zn_4Sb_3 EFAS sample (**Fig. S31**). The grain surface of the $Zn_{3.96}Cd_{0.04}Sb_3$ sample is very clean, although a small number of micro-cracks is still observed.

In the case of the $Zn_{3.97}Ge_{0.03}Sb_3$ sample, the surface color shows almost no change after the electromigration experiment (**Fig. S32**). Moreover, no cracking is observed in the $Zn_{3.97}Ge_{0.03}Sb_3$ sample, and the grain surfaces are really clean.

The above experimental observations show that the chemical stability of the

samples prepared by the EFAS method is greatly improved. Combined with doping at the Zn sites, particularly when doping with Ge, the samples become stable under a range of DC electric fields.

Supplementary Note 6

Pinpointing the underlying reaction mechanism of Zn_4Sb_3: Single-phase *β*-Zn_4Sb_3 can be obtained within 60 s under a pulsed DC field. If the charging time is extended to 83 s, ZnSb forms at the upstream side of the sample, and Zn fibers precipitate at the downstream side of the sample (**Fig. S33**). When the die with the sample is inverted (turned upside down) and charging continues for additional 18 s, single-phase Zn_4Sb_3 re-emerges everywhere again (**Fig. S34**). Obviously, there is a temporal window for the phase formation of *β*-Zn_4Sb_3, beyond which the decomposition occurs in the presence of an electric field.

Due to the fast ion migration behavior of Zn^{2+}, We cannot prepare single-phase Zn_4Sb_3 in precisely 60 s every time, so we placed a Zn plate at the upstream side of the sample (**Fig. S35**), which would extend the single-phase time window to a range of 55-64 s, and greatly improve the reproducibility of the sample.

If the starting material, rather than elemental powders of Zn and Sb, is made of "Zn+3ZnSb", the single-phase *β*-Zn_4Sb_3 compound can still be prepared under the pulsed electric current field within 30s (**Fig. S36**). The maximum reaction temperature detected in this case is only 420 K (**Fig. S37**), much lower than the growth-from-the-melt temperature of 1023 K[1,2], and the temperature of about 700 K used in the direct SPS synthesis[7,8]. Thus, it can be seen that the special phase formation process is more closely related to the electric field.

Supplementary Note 7

Synthesis of Zn_4Sb_3 through thermal explosion: 4 g powder mixtures of Zn (5N, 200 mesh) and Sb (5N, 200 mesh) were prepared according to the molar ratio of 4.2 : 3. A slight excess of Zn is required to supplement the loss of Zn during thermal explosion at elevated temperatures. The mixture is ground in an agate mortar for 20 min, and the uniformly mixed raw material is transferred into a steel die and pressed

under 10 MPa for 5 min to obtain a pellet. The pellet is sealed in an evacuated quartz tube (Φ20 mm), and the tube is subsequently placed into a furnace heated to 773 K for different times.

If only the heat treatment is used, a nearly single-phase Zn_4Sb_3 compound is obtained by thermal explosion at 773 K for 10 min (**Fig. S38**). The experiment indirectly indicates that the electric field-assisted synthesis technique of the Zn_4Sb_3 compound in a short period of time has a special formation mechanism, which is closely related to the DC current.

Interestingly, the Bi_2Te_3 single phased compound cannot be obtained in 60 s by using the EFAS method, along with residual Bi and Te in the product (**Fig. S39**). In the process, the peak temperature reached 541 K, only 3 K lower than the melting point of Bi (**Fig. S40**). If we continue to extend the reaction time or increase the current, bismuth will become liquid, flow out of the mold, and we will not get a single-phase compound any more. However, as shown previously, Bi_2Te_3 can be prepared rapidly by the self-propagating high-temperature synthesis technique in several seconds[9,10]. This indirectly proves that the EFAS method is related to the ion migration behavior.

Supplementary Note 8

Phase and morphology: Phase purity of all samples was inspected by X-ray powder diffraction (Empyrean, Cu K line, PANalytical, Holland). Images of freshly fractured surfaces were taken by field emission scanning electron microscopy (FESEM) (SU8000, Hitachi, Japan) with energy-dispersive X-ray spectroscopy (EDS) (XFlash6160, BRUKER, Germany). Back-scattered images were taken by EPMA (JXA-8100, JEOL, Japan). A direct characterization of the samples' atomic structures and structural evolution during the process of electrification was carried out on transmission electron microscopy (Talos F200s, FEI) and double CS-corrected transmission electron microscopy (Titan Themis G2 60-300, FEI). The samples for TEM observation were prepared by ion milling with liquid nitrogen (PIPS 695, Gatan) and focused ion beam (FIB) milling (Helios Nanolab G3 UC, FEI). *In situ* biasing experiments were performed within the TEM column using a TEM-STM holder (Pico Femto, ZEPTOOLS).

Fig. S41 shows in detail the intermediate stages in the phase transformation

process of "4Zn + 3Sb" mixed powders under the pulsed current. The ion transport channel in Zn_4Sb_3 facilitates fast mass transfer that participates in the chemical reaction.

Fig. S42 displays the fracture surface morphology and composition distribution of Zn_4Sb_3 prepared by the EFAS method. The sample is well compacted with a relative density higher than 98%, and the grain size on the scale of microns. The consistent composition contrast image documents the high phase purity of the final product.

Fig. S45 displays different stages (in time) during the current flow through β-Zn_4Sb_3 (MQ + SPS) via the *in situ* electron microscopy. As long as the voltage is applied, the grains will crack and disintegrate continually, and there is no phenomenon of grain boundary widening.

Supplementary Note 9

Heat flow tests: Fig. S43 and **S44** present the low and high temperature heat flow of the Zn_4Sb_3-based samples, respectively, detected by Q2000 (TA, USA).

For the Zn_4Sb_3 (MQ + SPS) compound in the low temperature region, there are two endothermic peaks on the heating stage (**Fig. S43**), corresponding to the temperatures of 236.6 K and 252.8 K, which correspond to phase transitions of α'-α and α-β, respectively. Because the phase transition process is reversible, there are two exothermic peaks on the cooling stage, corresponding to temperatures of 234.4 K and 250.1 K. This may explain why the carrier concentration fluctuates violently near 230K (**Fig. S6c**). In the high temperature region, the Zn_4Sb_3 (MQ + SPS) compound exhibits a broad exothermic peak around 428 K (at 417.7 K and 438.3 K) and an endothermic peak at 561.5 K (**Fig. S44a**), indicating that the sample is unstable. This corresponds to the sharp fluctuation of the carrier concentration near 423 K and 560 K (**Fig. S5a**). This phenomenon was also observed by other authors. Lin *et al.*[11] found that Zn_4Sb_3 compound becomes metastable and gradually decomposes into Zn(hcp) and ZnSb around 425 K, while it recovers its stability above 565 K.

More interestingly, in the low temperature region, there is only one endothermic peak on the heating stage and one exothermic peak on the cooling stage of the Zn_4Sb_3 sample synthesized by the EFAS method, corresponding to the temperature of 235.4 K and 232.9 K (**Fig. S43**). With the increasing content of Cd doping at the site of Zn, the latent heat gradually decreases (**Fig. S43a**). In the high temperature region, there

is no obvious heat absorption and the release peak in the heat flow curve of Cd-doped samples, only a slight fluctuation near 428 K and 560 K (**Fig. S44c**). The above observed phenomena show that Cd doping, combined with the EFAS method has a significant impact on the low temperature phase transition process, and can, to a certain degree, stabilize the high temperature structure.

In lightly Ge-doped samples, the position of the endothermic peak is significantly lower than that in the undoped sample in the low temperature range (**Fig. S43b**). The endothermic peak is reduced to 222 K and 215 K for 0.25at% and 0.5at% Ge-doping samples, respectively, and the latent heat of phase transition is greatly reduced. When the doping content of Ge is 0.75at%, there is almost no phase transition peak observed in the range of 203 K- 313 K. The high temperature heat flow curve of the 0.25at% Ge-doped sample still fluctuates slightly near 428 K and 560 K (**Fig. S44e**), and the high temperature heat flow curves become very flat when the doping content of Ge increases further. The above observed experimental results show that Ge doping can greatly inhibit the low temperature phase transition and stabilize the high temperature structure of the material.

Surprisingly, all samples, including Zn_4Sb_3 (MQ + SPS), $(Zn_{1-x}Cd_x)_4Sb_3$ (EFAS) and $(Zn_{1-x}Ge_x)_4Sb_3$ (EFAS), are still single-phase β-Zn_4Sb_3 structure after the DSC cycle test with the highest temperature of 673 K (**Fig. S44**). From this point of view, Zn_4Sb_3 can be re-synthesized on the cooling cycle.

Supplementary Note 10

Other compounds obtained through EFAS technique: More interestingly, the high TE performance ZnSb compound could also be synthesized within 60 s by the EFAS process. **Fig. S46** shows the reaction parameters, phase composition, and the corresponding TE performance. At the same time, we subdivided the synthesis process, and the intermediate stages in the phase transformation process of "Zn + Sb" mixed powder under the pulsed current are displayed in **Fig. S47**.

In the process, the highest temperature of the stoichiometric "Zn + Sb" mixed powder is only 514 K. As is well known, the ZnSb compound is not a fast ion conductor, so how come that it can also be synthesized so quickly? It is precisely because of the formation of the Zn_4Sb_3 compound during the intermediate reaction process that the ion transport channels of Zn formed. This leads to rapid chemical reactions and the synthesis of ZnSb in such mild conditions.

The ZnSb compound synthesized by this technique has a relatively high TE performance (**Fig. S46**). In the range of 300K - 723 K, the electrical conductivity σ decreases at first and then increases with the temperature, while the Seebeck coefficient α increases at first and then decreases correspondingly. There is a turning point near 450 K, which may be the result of intrinsic excitations. The power factor *PF* can reach 1.35 $mWm^{-1}K^{-2}$ at 723K. With the rising temperature, the overall thermal conductivity κ decreases at first and then increases, and the lowest value of 1.16 $Wm^{-1}K^{-1}$ is reached at 550 K. Due to the low σ of the material, the lattice thermal conductivity κ_L accounts for the vast majority of the total thermal conductivity. Finally, the calculated *ZT* value reaches its maximum of 0.71 at 727 K.

Moreover, we also tried to prepare Cu_2Se and Cu_2S compounds using the EFAS process. **Fig. S48** displays the reaction parameters and phase composition of stoichiometric "2Cu + Se" and "2Cu + S" mixed powders under the pulsed electric field. It only takes 30 s to synthesize Cu_2Se and Cu_2S compounds, and the highest detectable temperatures are only 370 K and 354 K, respectively.

The above experiments show that the electric field-assisted synthesis method, by relying on the ion transport channels to achieve rapid mass transfer, may be suitable for the synthesis of solid fast ionic conductor materials or compounds.

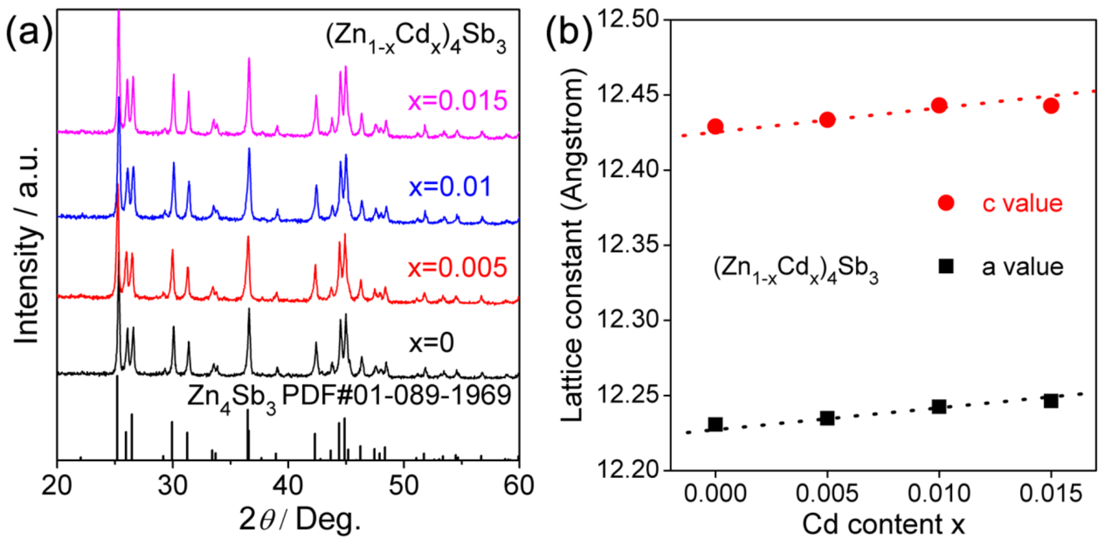

Supplementary Figure 1. (a) XRD patterns of compounds with different Cd-contents and (b) the lattice parameters *a* and *c* as a function of Cd-substituted content.

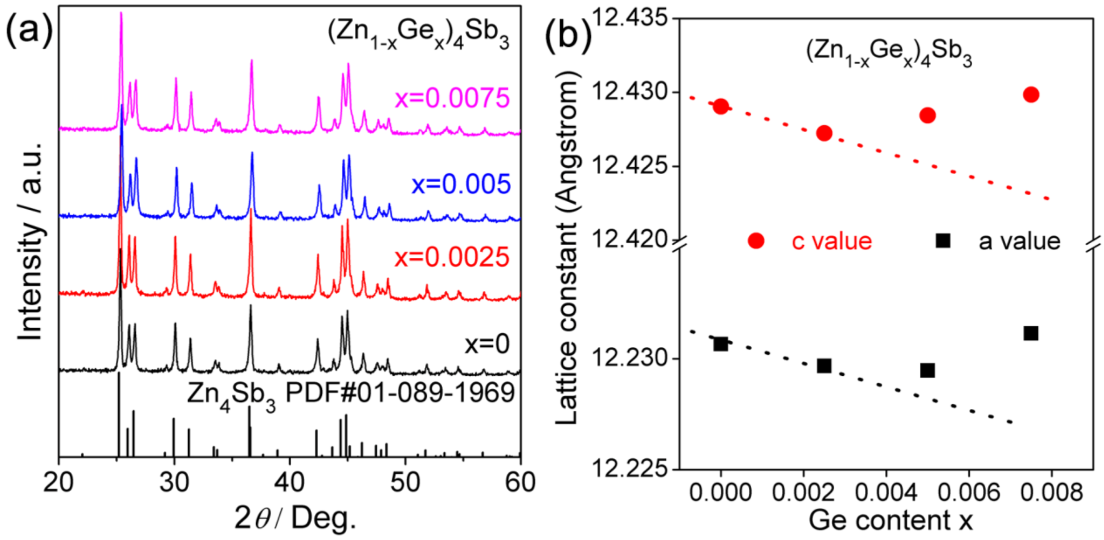

Supplementary Figure 2. (a) XRD patterns of compounds with different Ge-contents and (b) the lattice parameters *a* and *c* as a function of Ge-substituted content.

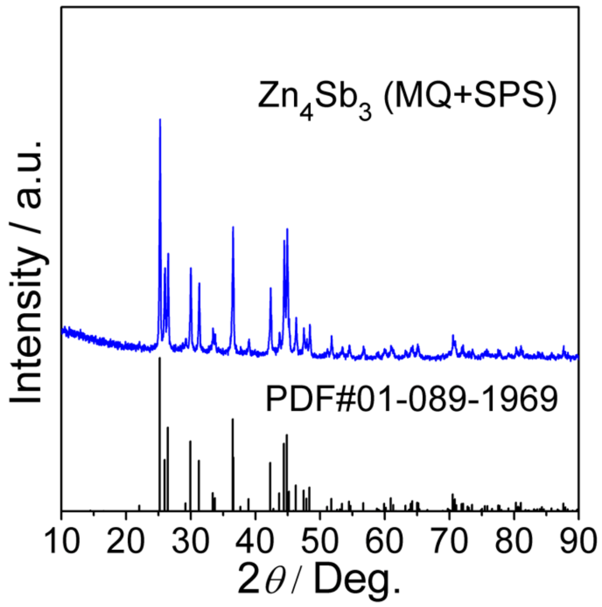

Supplementary Figure 3. Phase composition of the Zn_4Sb_3 (MQ + SPS) sample synthesized by melting at high temperature, followed by quenching in salt water, then consolidated by spark plasma sintering (SPS).

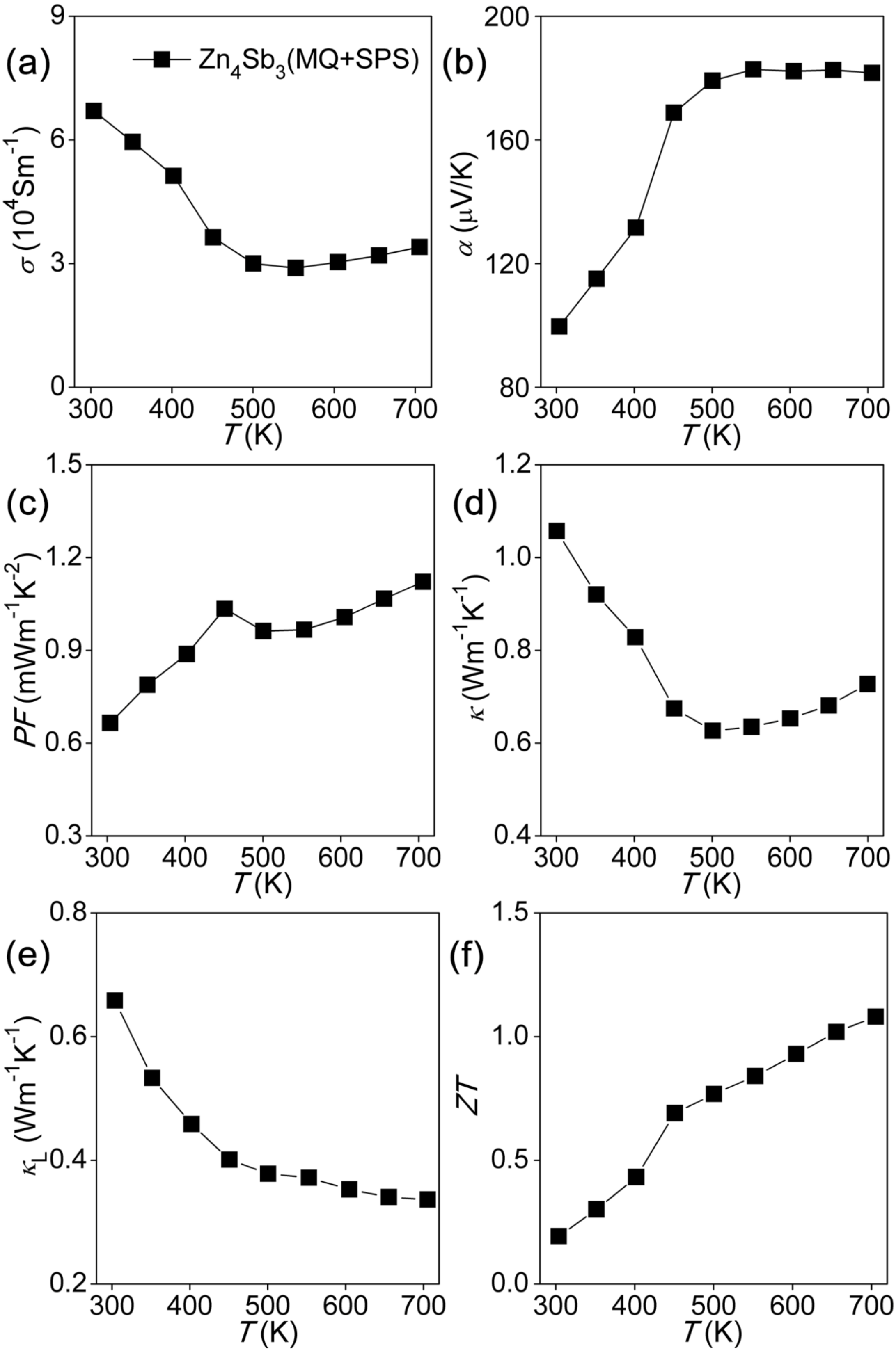

Supplementary Figure 4. Thermoelectric performance of the Zn_4Sb_3(MQ + SPS) compound: (a) electrical conductivity, (b) Seebeck coefficient, (c) power factor, (d) total thermal conductivity, (e) lattice thermal conductivity, (f) Fig. of merit *ZT*.

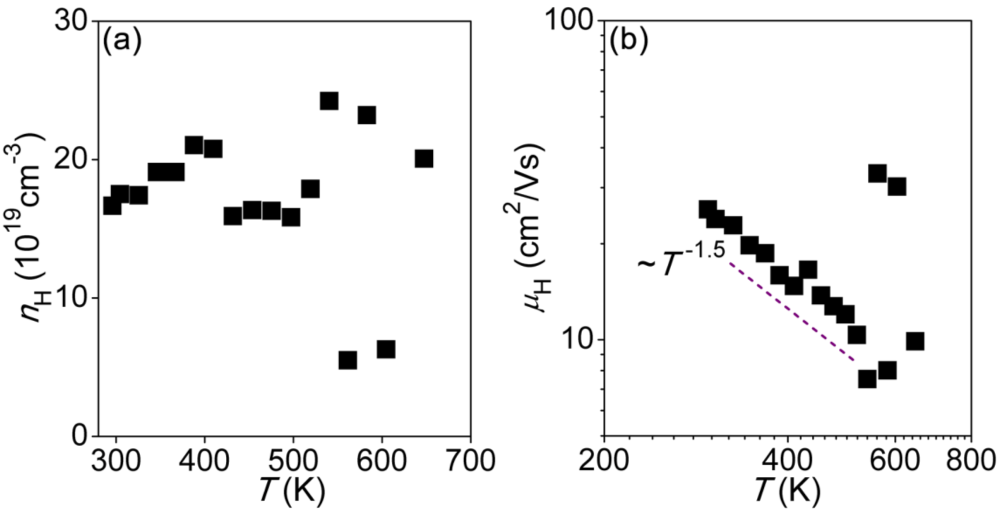

Supplementary Figure 5. High temperature electronic transport properties of the Zn_4Sb_3 (MQ + SPS) compound: (a) carrier concentration, (b) carrier mobility.

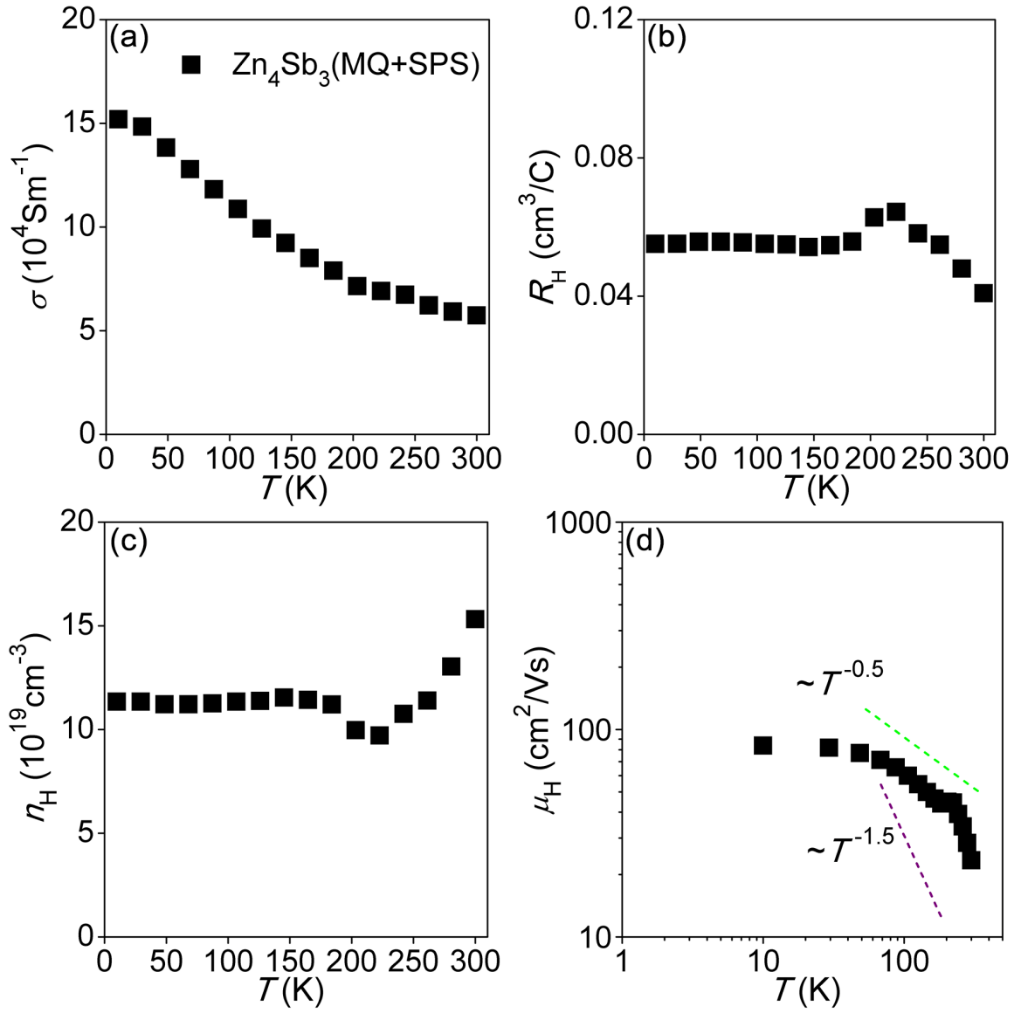

Supplementary Figure 6. Low temperature electronic transport properties of the Zn_4Sb_3 (MQ + SPS) compound: (a) electrical conductivity, (b) Hall factor, (c) carrier concentration, (d) carrier mobility.

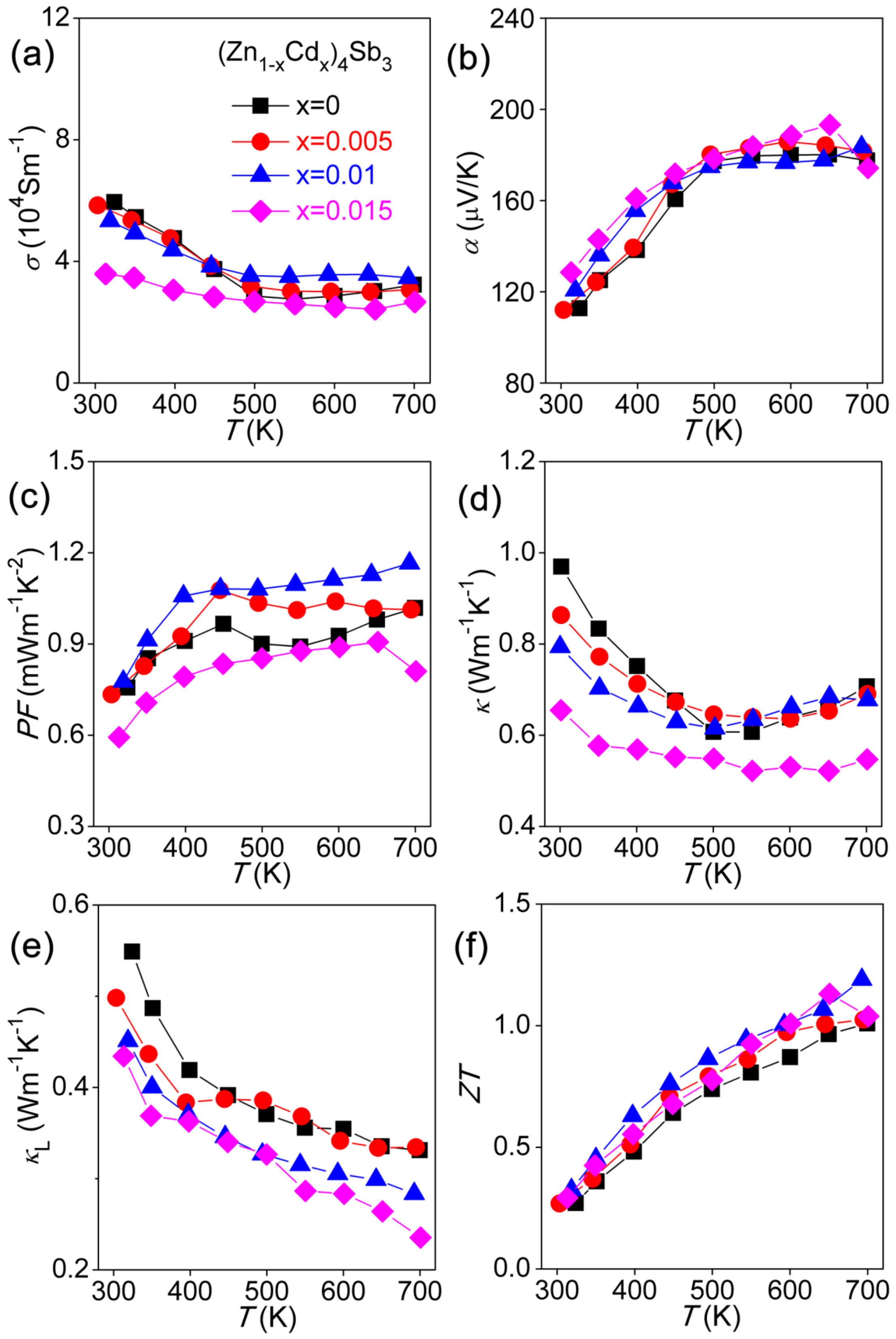

Supplementary Figure 7. Thermoelectric performance of $(Zn_{1-x}Cd_x)_4Sb_3$ (EFAS) compounds: (a) electrical conductivity, (b) Seebeck coefficient, (c) power factor, (d) total thermal conductivity, (e) lattice thermal conductivity, (f) Fig. of merit *ZT*.

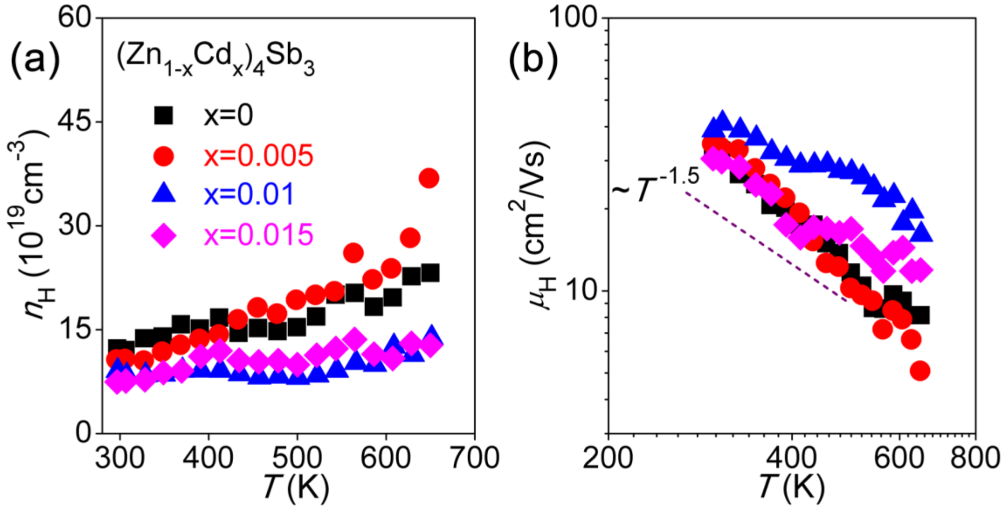

Supplementary Figure 8. High temperature electronic transport properties of $(Zn_{1-x}Cd_x)_4Sb_3$ (EFAS) compounds: (a) carrier concentration, (b) carrier mobility.

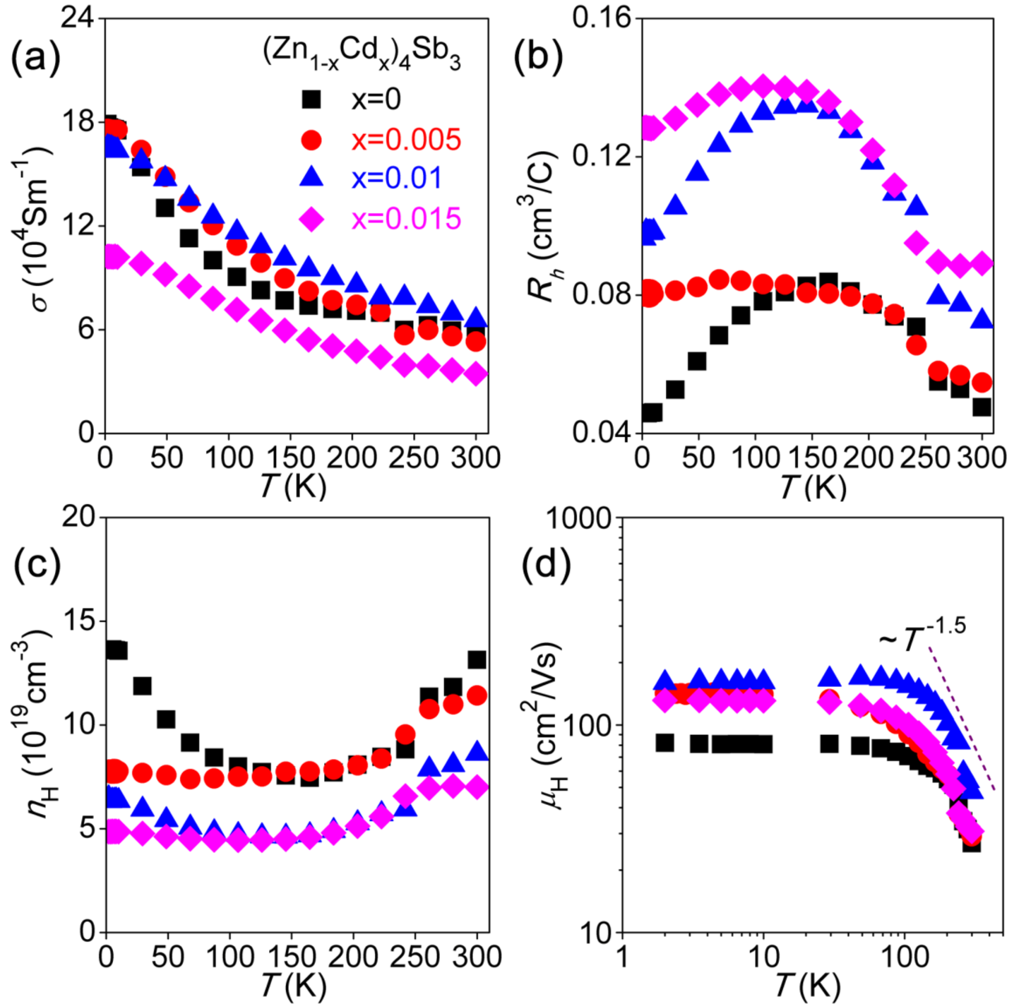

Supplementary Figure 9. Low temperature electronic transport properties of $(Zn_{1-x}Cd_x)_4Sb_3$ (EFAS) compounds: (a) electrical conductivity, (b) Hall factor, (c) carrier concentration, (d) carrier mobility.

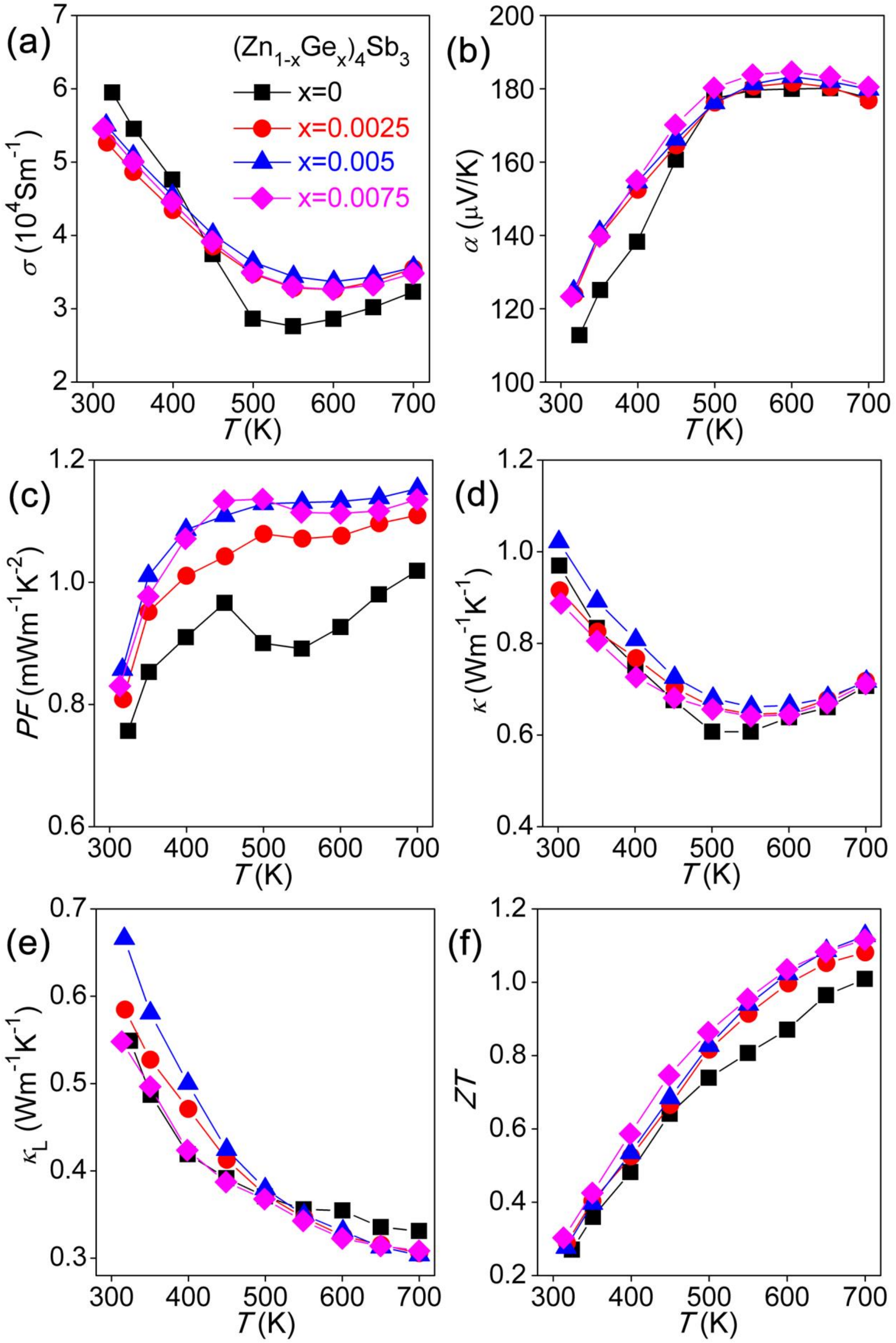

Supplementary Figure 10. Thermoelectric performance of $(Zn_{1-x}Ge_x)_4Sb_3$ (EFAS) compounds: (a) electrical conductivity, (b) Seebeck coefficient, (c) power factor, (d) total thermal conductivity, (e) lattice thermal conductivity, (f) Fig. of merit *ZT*.

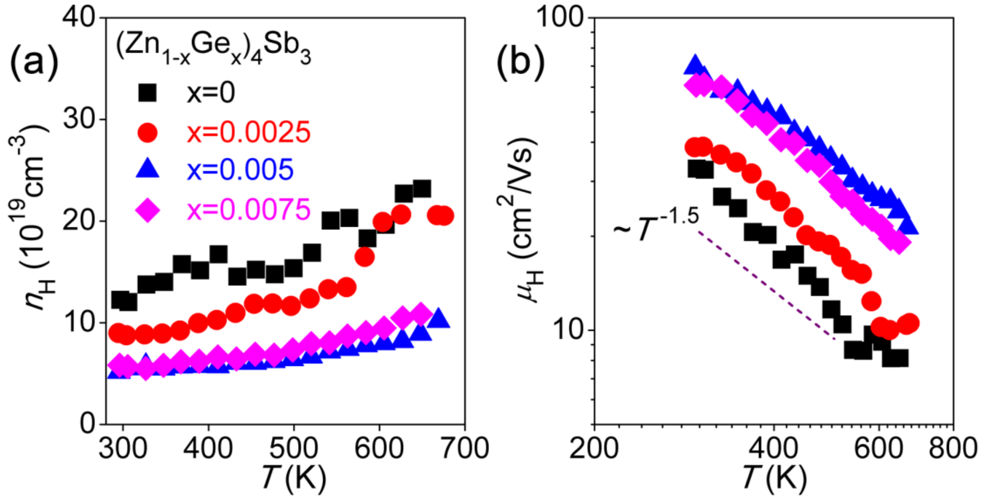

Supplementary Figure 11. High temperature electronic transport properties of $(Zn_{1-x}Ge_x)_4Sb_3$ (EFAS) compounds: (a) carrier concentration, (b) carrier mobility.

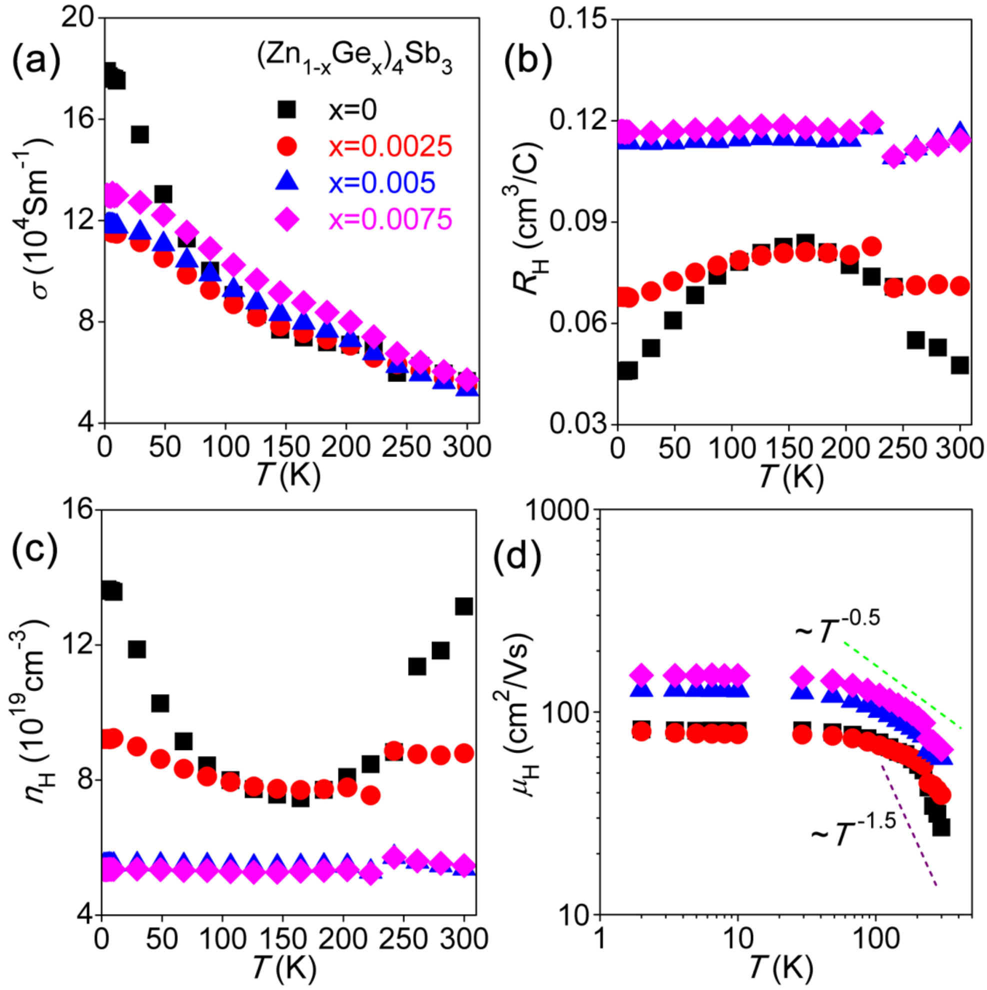

Supplementary Figure 12. Low temperature electronic transport properties of $(Zn_{1-x}Ge_x)_4Sb_3$ (EFAS) compounds: (a) electrical conductivity, (b) Hall factor, (c) carrier concentration, (d) carrier mobility.

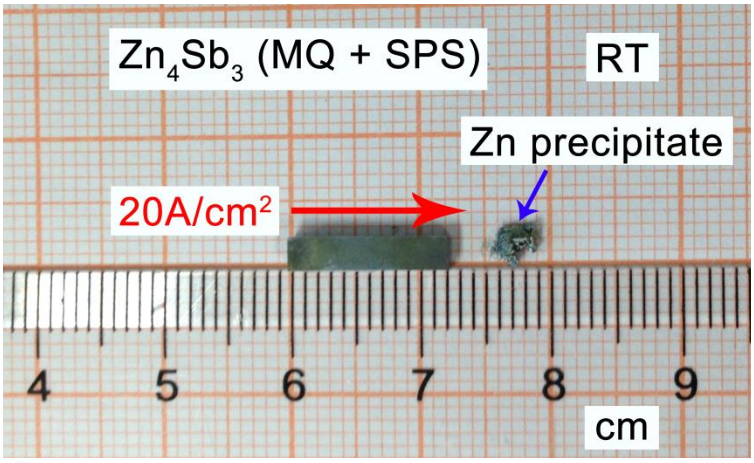

Supplementary Figure 13. Chemical electromigration experiment on the Zn_4Sb_3 (MQ + SPS) bulk sample was conducted at ambient conditions for one day with the DC current density of 20 A/cm^2.

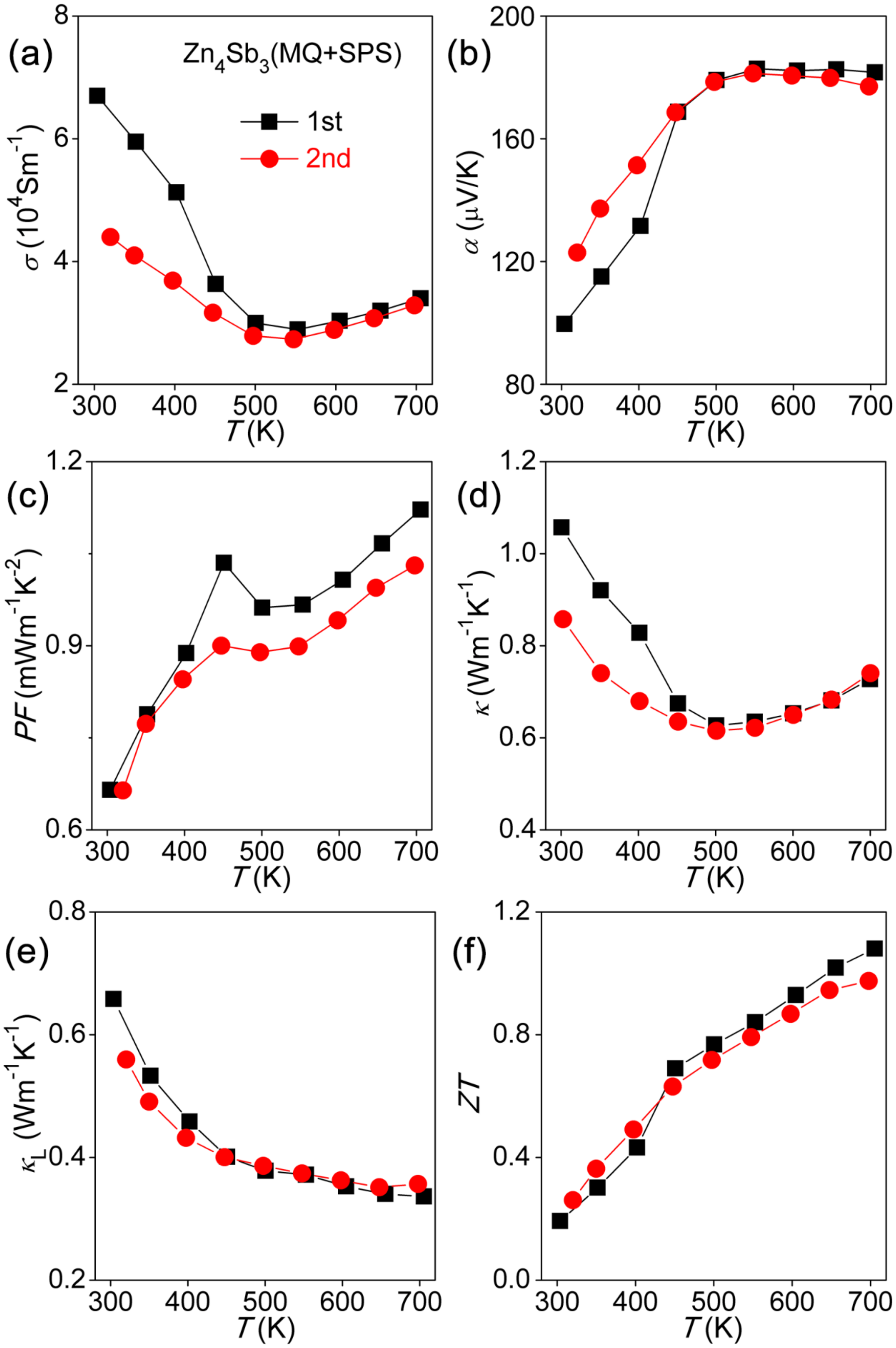

Supplementary Figure 14. High temperature thermoelectric performance of the Zn_4Sb_3 (MQ + SPS) sample in two cycle test.

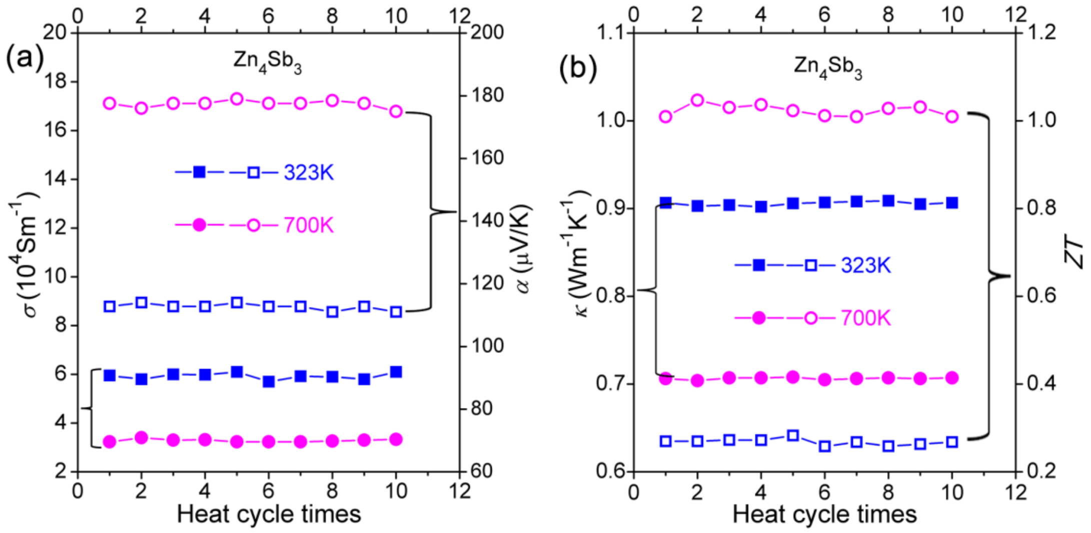

Supplementary Figure 15. Thermoelectric performance of the Zn_4Sb_3 (EFAS) sample after 10 cycle testing between 323 K and 700 K, (a) electrical conductivity and Seebeck coefficient, (b) total thermal conductivity and *ZT* values.

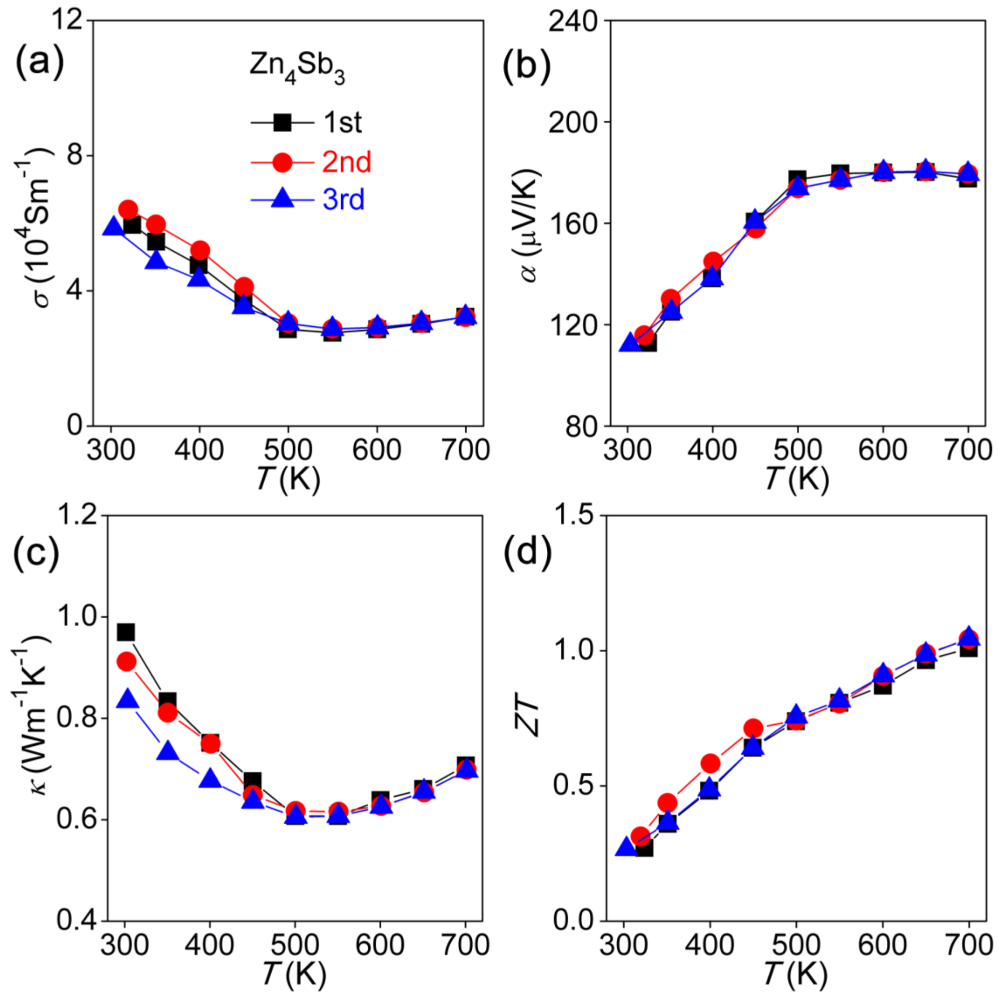

Supplementary Figure 16. Thermoelectric performance of three Zn_4Sb_3 (EFAS) samples prepared in different batches, (a) electrical conductivity, (b) Seebeck coefficient, (c) total thermal conductivity, (d) *ZT* values.

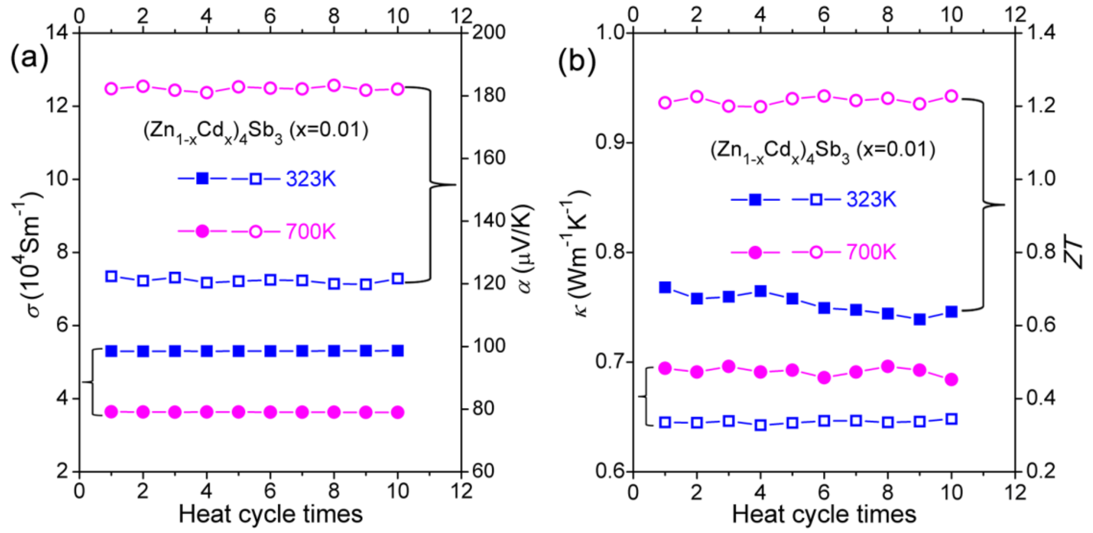

Supplementary Figure 17. Thermoelectric performance of the $Zn_{3.96}Cd_{0.04}Sb_3$ (EFAS) sample after 10 cycle testing between 323 K and 700 K, (a) electrical conductivity and Seebeck coefficient, (b) total thermal conductivity and *ZT* values.

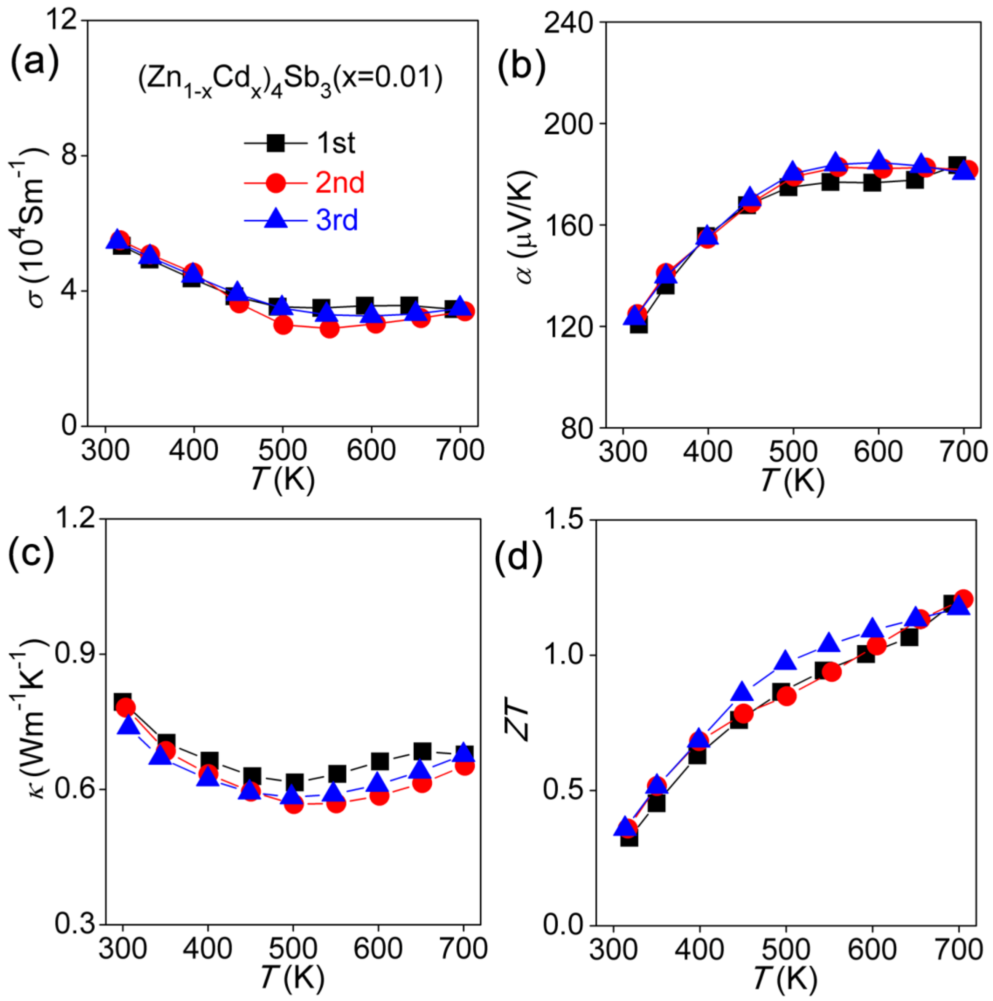

Supplementary Figure 18. Thermoelectric performance of three $Zn_{3.96}Cd_{0.04}Sb_3$ (EFAS) samples prepared in different batches, (a) electrical conductivity, (b) Seebeck coefficient, (c) total thermal conductivity, (d) *ZT* values.

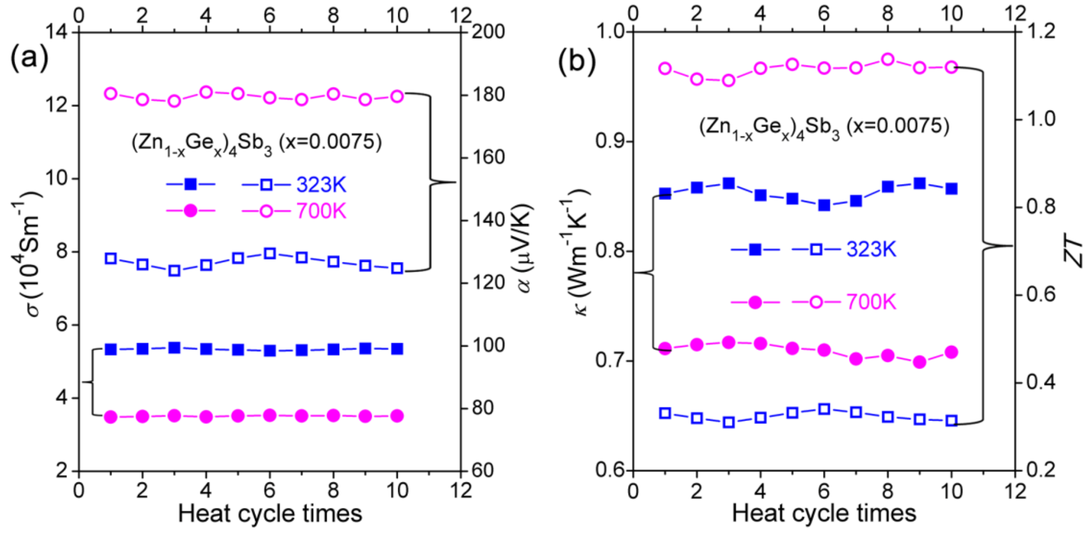

Supplementary Figure 19. Thermoelectric performance of the $Zn_{3.97}Ge_{0.03}Sb_3$ (EFAS) sample after 10 cycle testing between 323 K and 700 K, (a) electrical conductivity and Seebeck coefficient, (b) total thermal conductivity and *ZT* values.

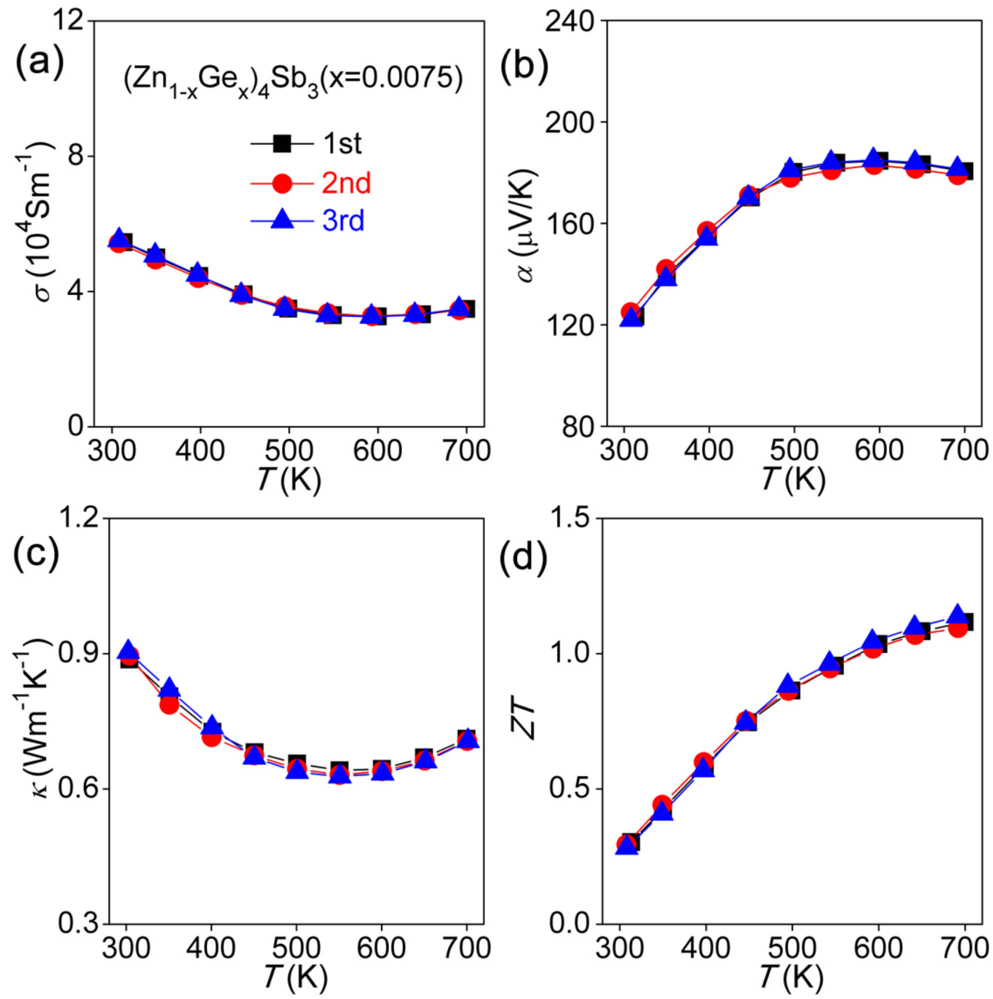

Supplementary Figure 20. Thermoelectric performance of three $Zn_{3.97}Ge_{0.03}Sb_3$ (EFAS) samples prepared in different batches, (a) electrical conductivity, (b) Seebeck coefficient, (c) total thermal conductivity, (d) *ZT* values.

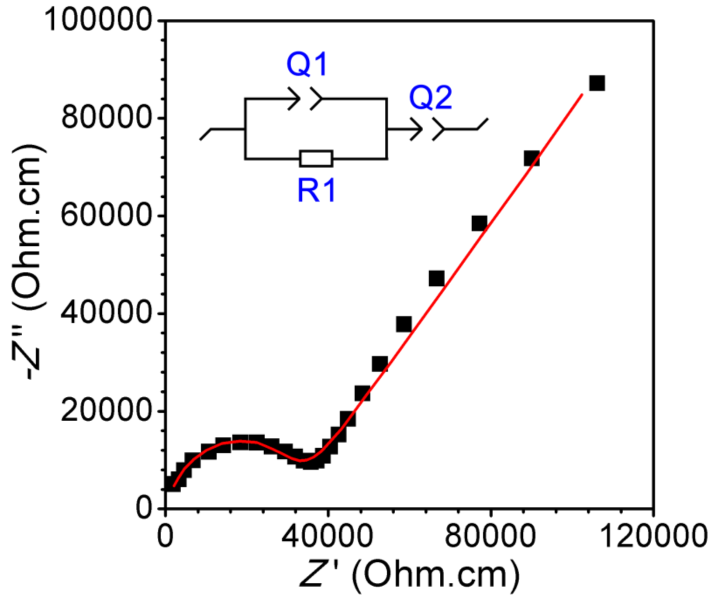

Supplementary Figure 21. Impedance plot (100 Hz - 5 MHz) of zinc-loaded montmorillonite measured under ambient conditions. The solid line represents simulated data with an equivalent circuit consisting of (Q1R1)(Q2) (where R1 is the ion resistance, Q is the constant phase element， and Q1 and Q2 refer to conduction of the grain and interface diffusion, respectively.

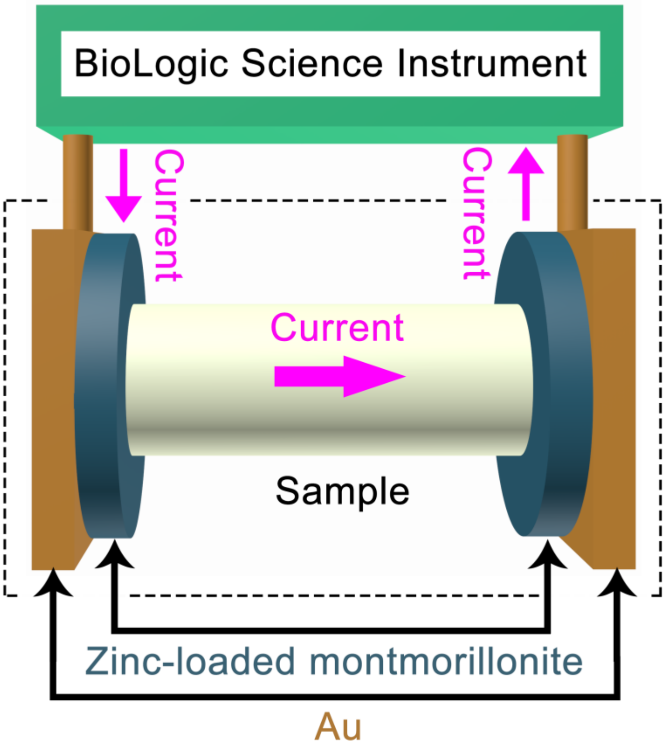

Supplementary Figure 22. Schematic diagram of a customer-designed device for the ionic conductivity measurement. It is based on the electron-blocking method. Two ionic Au|zinc-loaded montmorillonite electrodes are used to connect with the two ends of Zn_4Sb_3-based compounds to form a Au|zinc-loaded montmorillonite|Zn_4Sb_3 based compounds|zinc-loaded montmorillonite|Au pseudo-galvanic cell. These ionic electrodes can block the holes passing across the Zn_4Sb_3-based compounds|zinc-loaded montmorillonite interfaces while allowing the ions to migrate freely through them. Meanwhile, two ionic Au|zinc-loaded montmorillonite probes are used to record the potential variation on the sample generated by the movable ions. The total voltage drop is the sum over zinc-loaded montmorillonite and the Zn_4Sb_3-based compounds. When the ionic conductivity of zinc-loaded montmorillonite is known, the ionic conductivity of the Zn_4Sb_3-based compounds can be obtained. Meanwhile, all samples have similar sizes of $8 \times 8 \times 1.3$ mm^3.

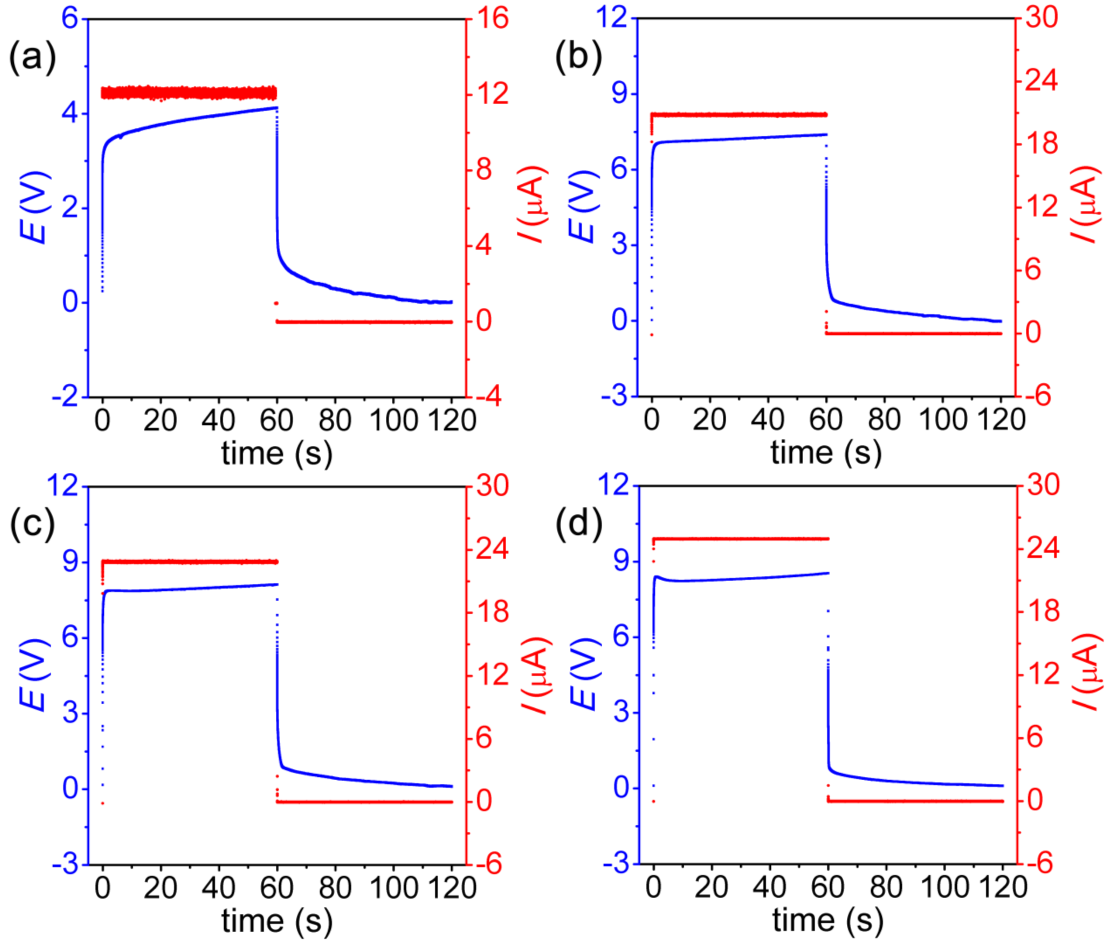

Supplementary Figure 23. Potential variation curves for Zn_4Sb_3 (MQ + SPS) under different test current at 298 K applied for 60 sec. (a) 12 μA, (b) 21 μA, (c) 23 μA, and (d) 25 μA.

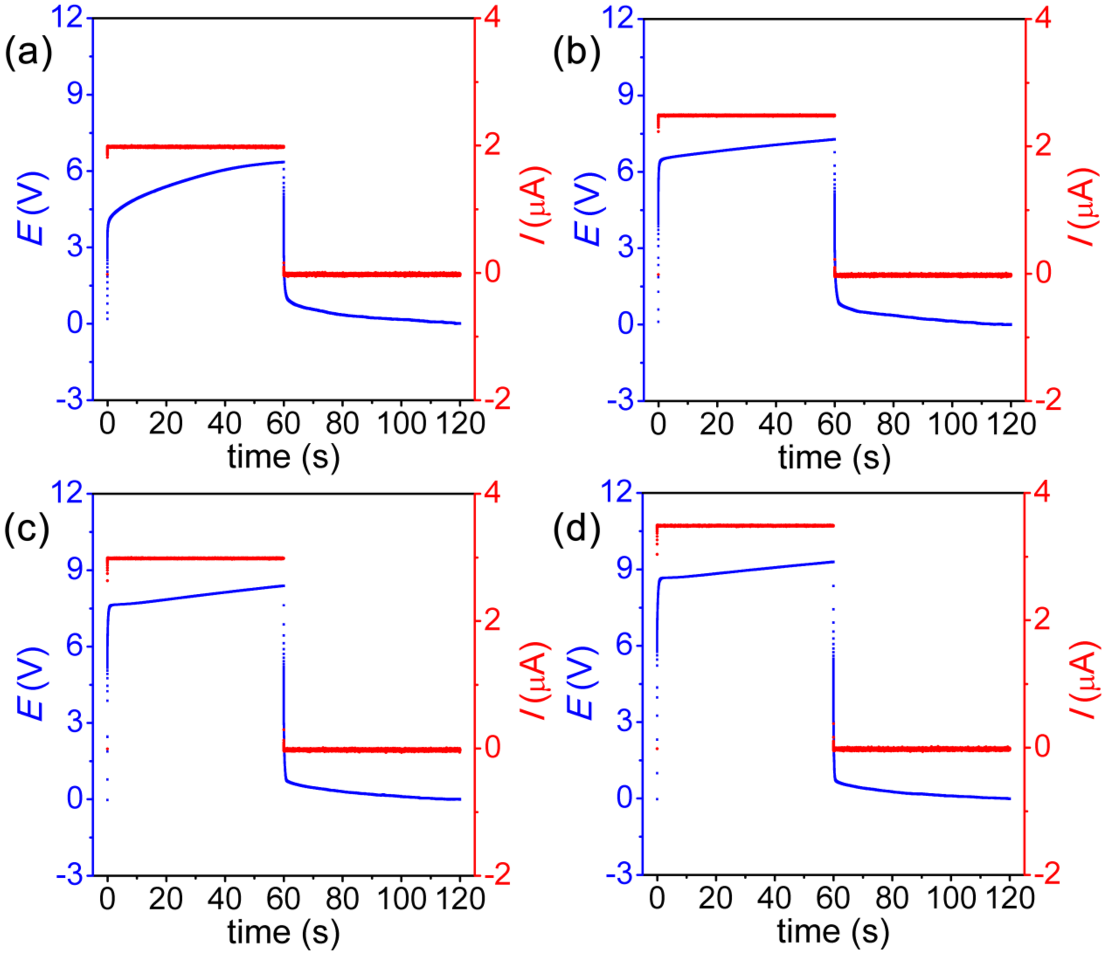

Supplementary Figure 24. Potential variation curves for Zn_4Sb_3 (EFAS) under different test current at 298 K applied for 60 sec. (a) 2.0 μA, (b) 2.5 μA, (c) 3.0 μA, and (d) 3.5 μA.

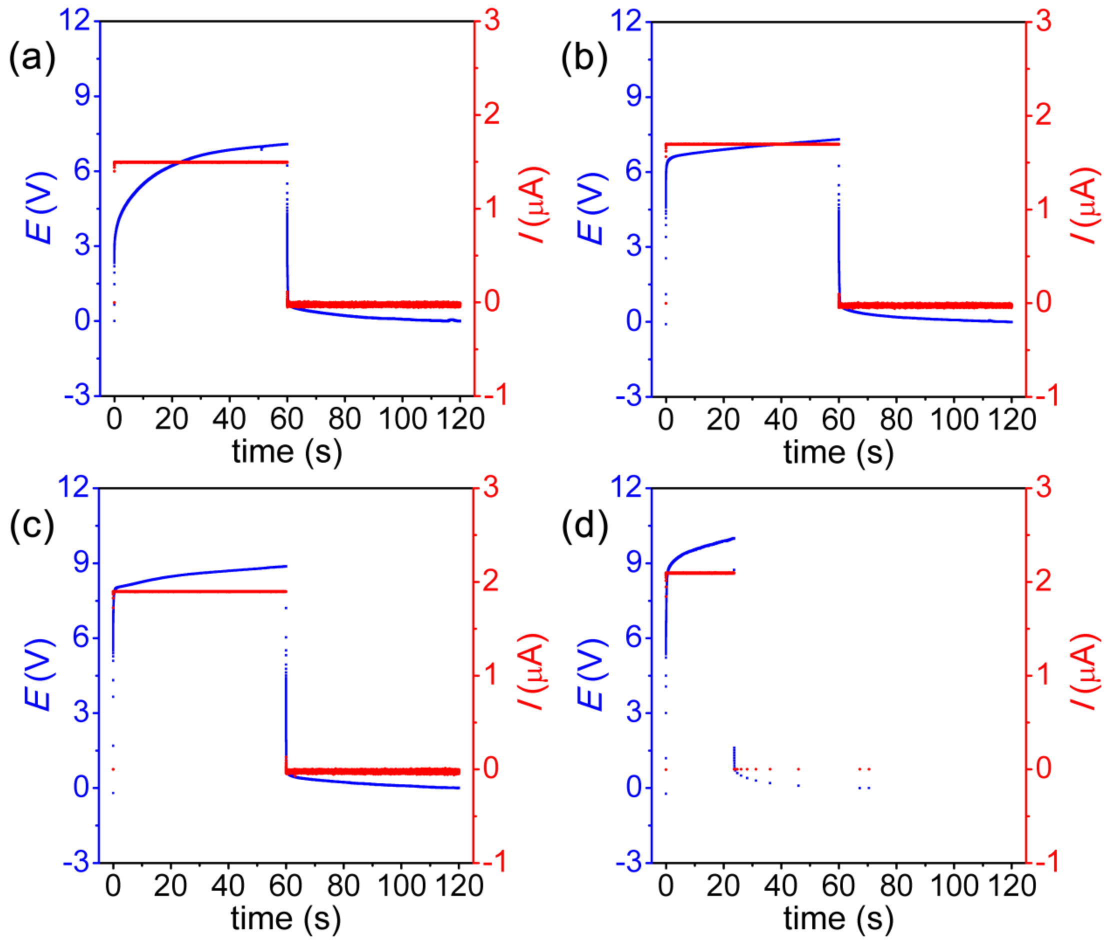

Supplementary Figure 25. Potential variation curves for $Zn_{3.96}Cd_{0.04}Sb_3$ (EFAS) under different test current at 298 K applied for 60 sec. (a) 1.5 μA, (b) 1.7 μA, (c) 1.9 μA, and (d) 2.1 μA.

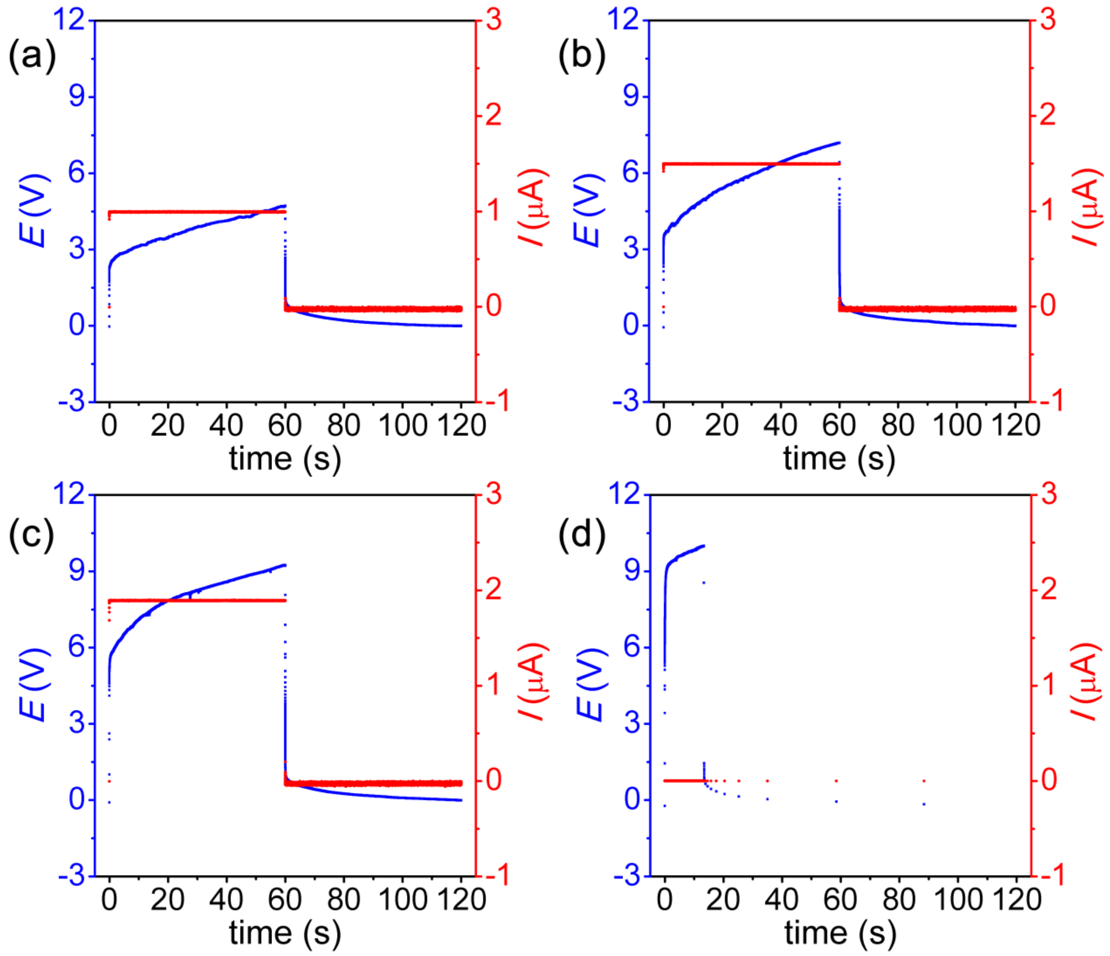

Supplementary Figure 26. Potential variation curves for $Zn_{3.97}Ge_{0.03}Sb_3$ (EFAS) under different test current at 298 K applied for 60 sec. (a) 1.0 μA, (b) 1.5 μA, (c) 1.9 μA, and (d) 2.1 μA.

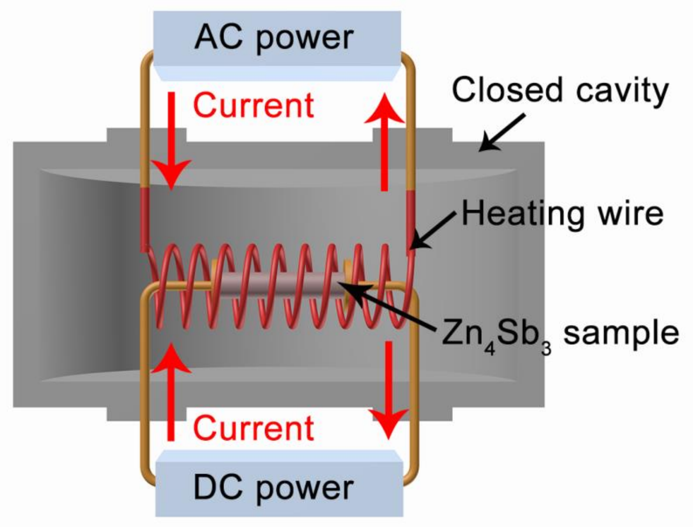

Supplementary Figure 27. Schematic diagram of the chemical electromigration experiment. The external heating wire provides a high temperature environment for the test sample being charged, and the two ends of the Zn_4Sb_3-based sample are charged with the direct current, and a certain amount of inert gas can be filled into the cavity to avoid a short circuit.

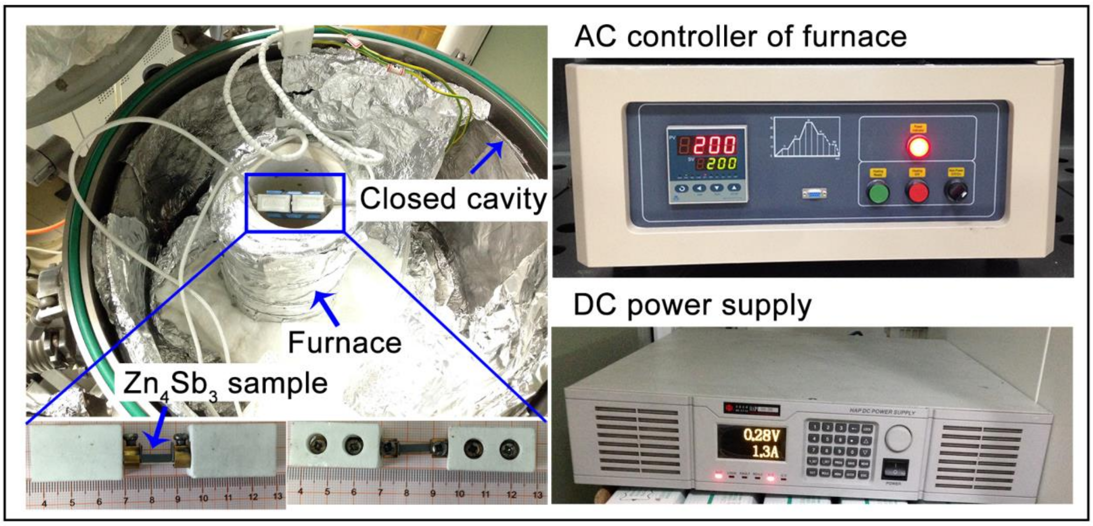

Supplementary Figure 28. Home-made chemical electromigration experimental device, the external AC power controller (KSL-1200X, HF-Kejing, China) controls the furnace temperature, DC power supply (HAP 30-30, Hua Tai, China) charges the test sample. In this experiment, the furnace temperature was set at 473 K, the DC current density was 20 A/cm^2, and the high purity argon gas of 100 Pa filled the cavity to avoid a short circuit.

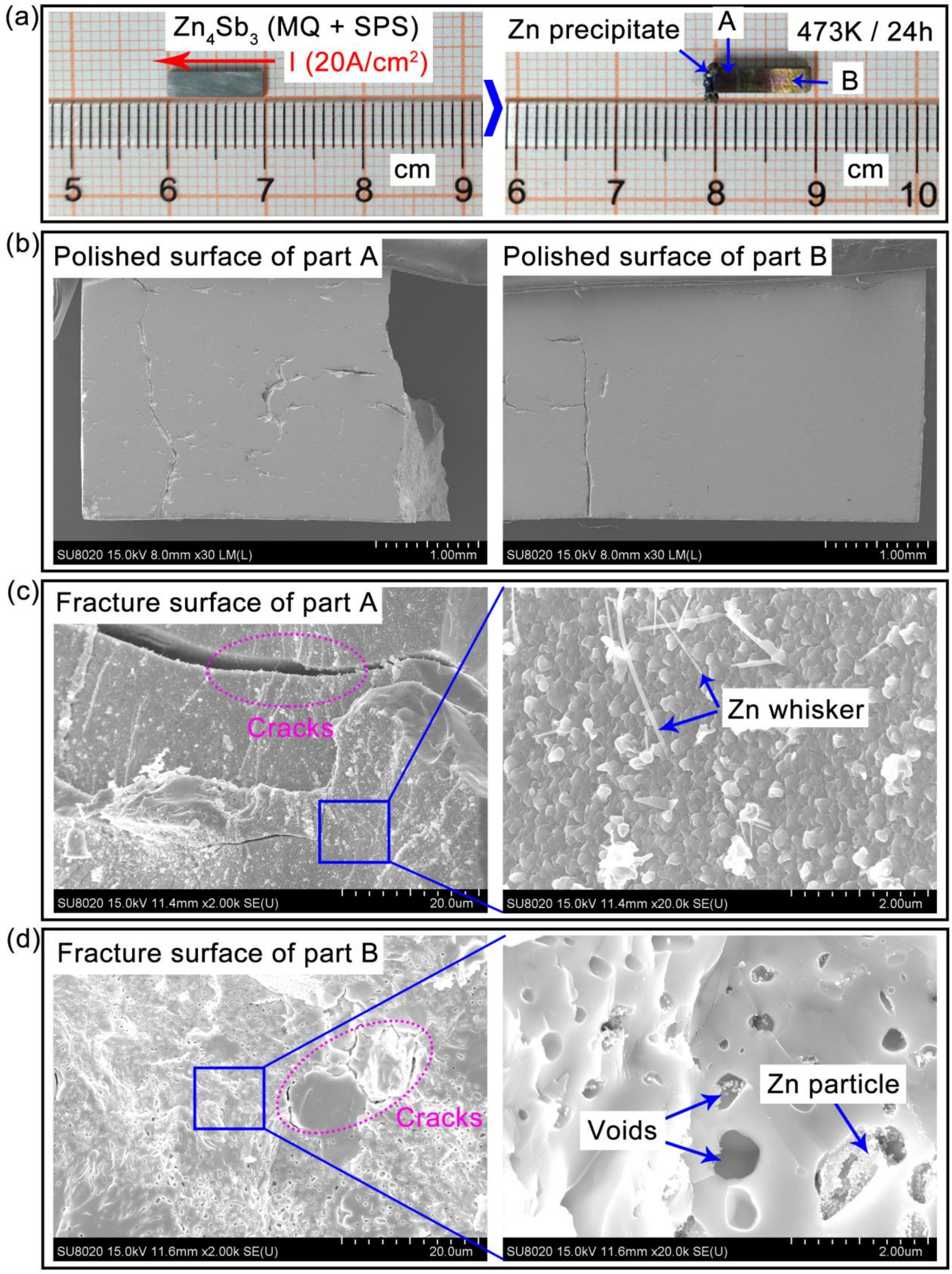

Supplementary Figure 29. Chemical electromigration experiment on the Zn_4Sb_3 (MQ + SPS) bulk sample. The environment temperature was 473 K, the DC current density was 20 A/cm^2, and the charging time was 24 h. (a) Sample morphology before and after the experiment, (b) low magnification morphology of A (downstream direction of current) and B (upstream direction of current) part of the sample after experiment, (c) fracture surface morphology of part A, (d) fracture surface morphology of part B.

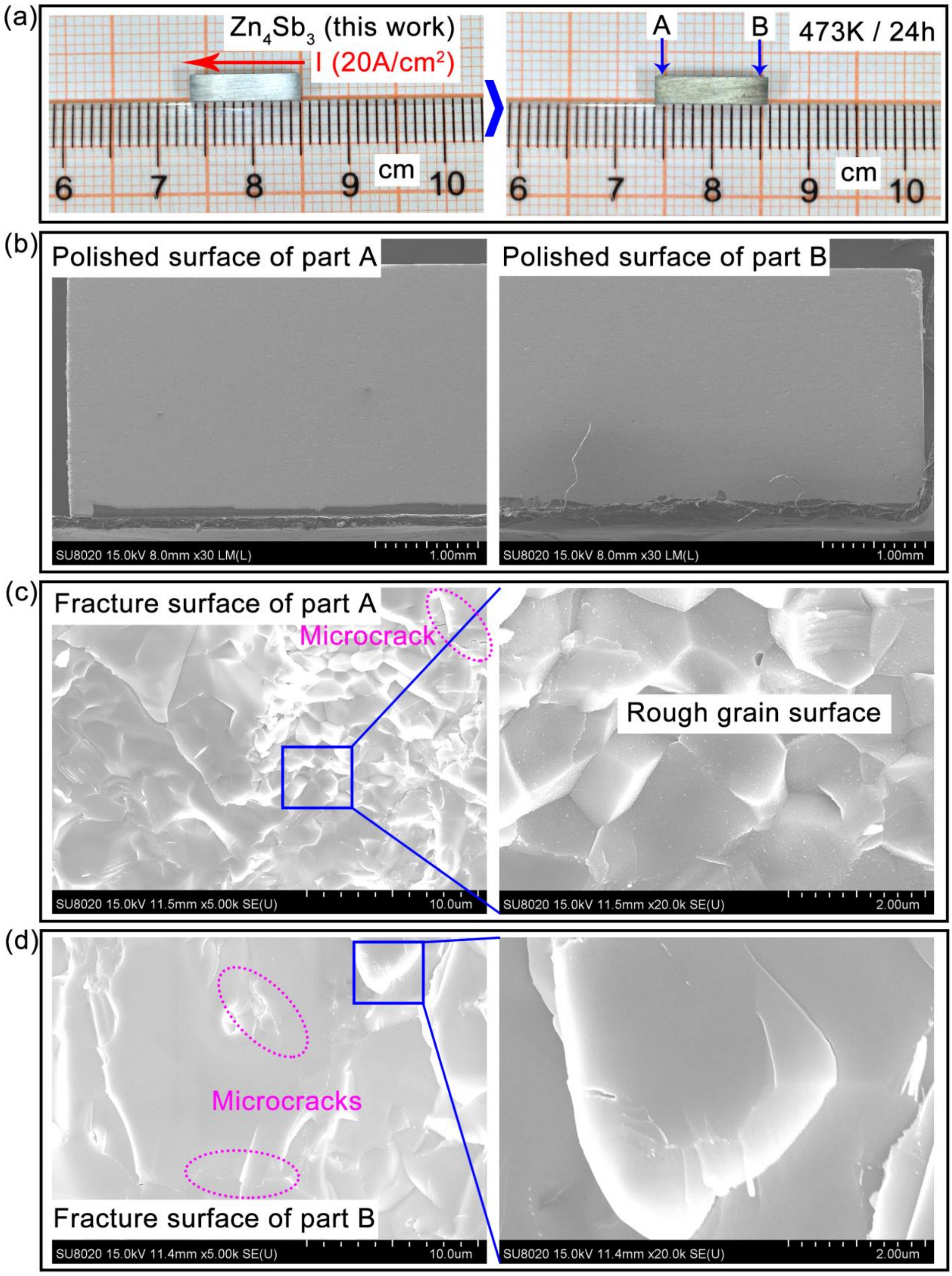

Supplementary Figure 30. Chemical electromigration experiment on the Zn_4Sb_3 bulk sample prepared by the EFAS method. The environment temperature was 473 K, the DC current density was 20 A/cm^2, and the charging time was 24 h. (a) Sample morphology before and after the experiment, (b) low magnification morphology of A (downstream direction of current) and B (upstream direction of current) part of the sample after experiment, (c) fracture surface morphology of part A, (d) fracture surface morphology of part B.

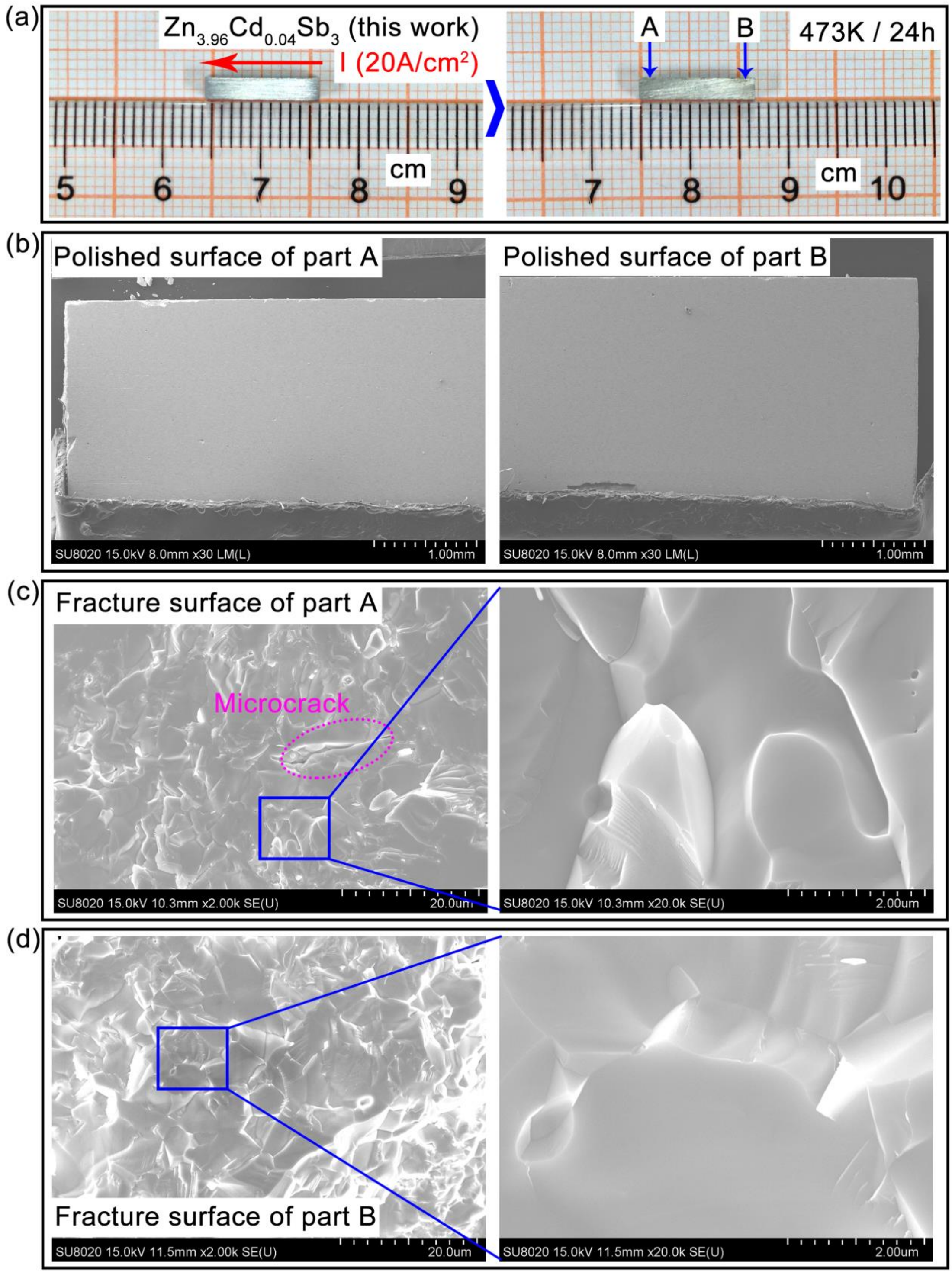

Supplementary Figure 31. Chemical electromigration experiment on the $Zn_{3.96}Cd_{0.04}Sb_3$ bulk sample prepared by the EFAS method. The environment temperature was 473 K, the DC current density was 20 A/cm^2, and the charging time was 24 h. (a) sample morphology before and after the experiment, (b) low multiple morphology of A (downstream direction of current) and B (upstream direction of current) part of the sample after experiment, (c) fracture surface morphology of part A, (d) fracture surface morphology of part B.

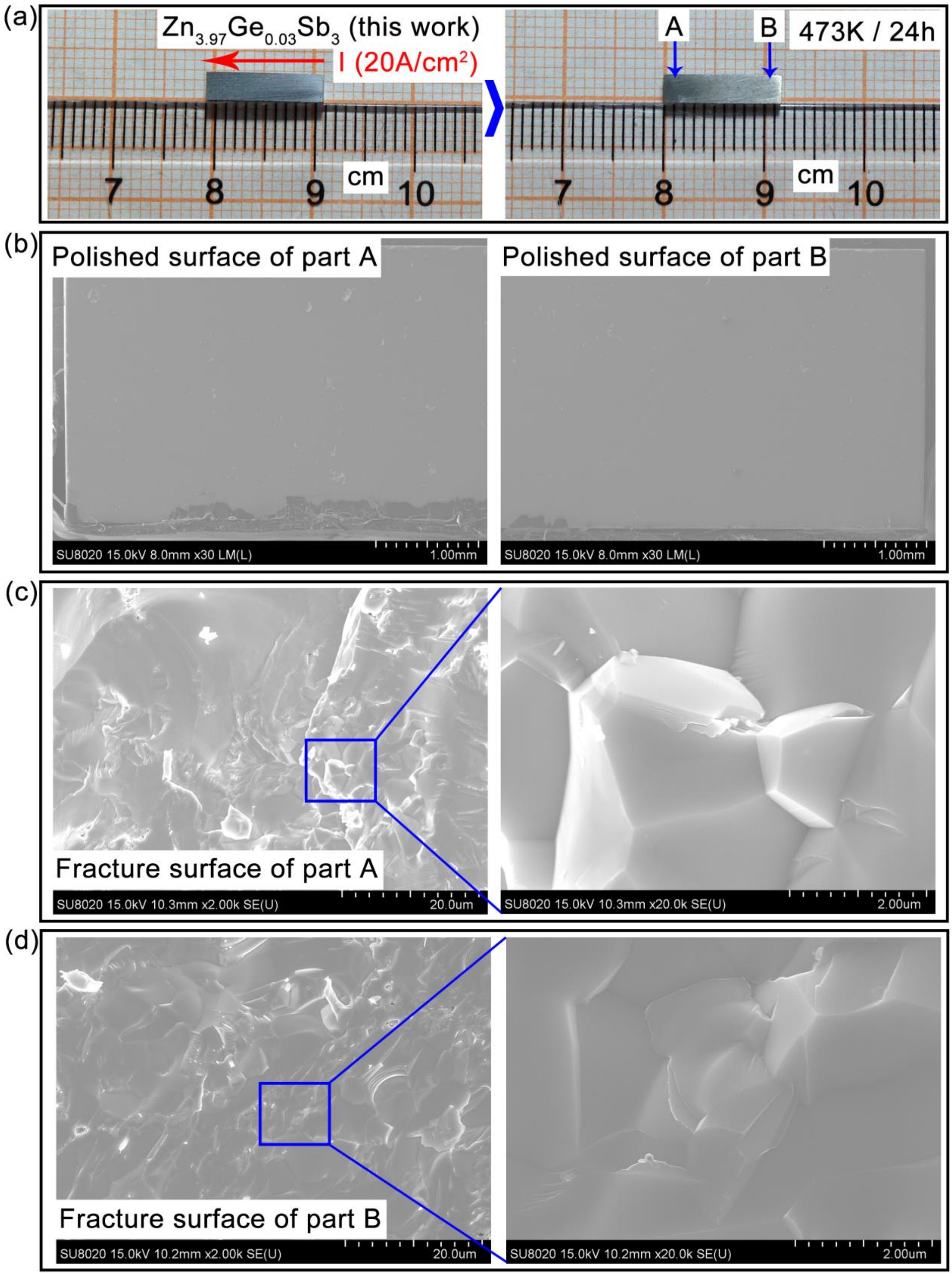

Supplementary Figure 32. Chemical electromigration experiment on the $Zn_{3.97}Ge_{0.03}Sb_3$ bulk sample prepared by the EFAS method. The environment temperature was 473 K, the DC current density was 20 A/cm^2, and the charging time was 24 h. (a) sample morphology before and after the experiment, (b) low multiple morphology of A (downstream direction of current) and B (upstream direction of current) part of the sample after experiment, (c) fracture surface morphology of part A, (d) fracture surface morphology of part B.

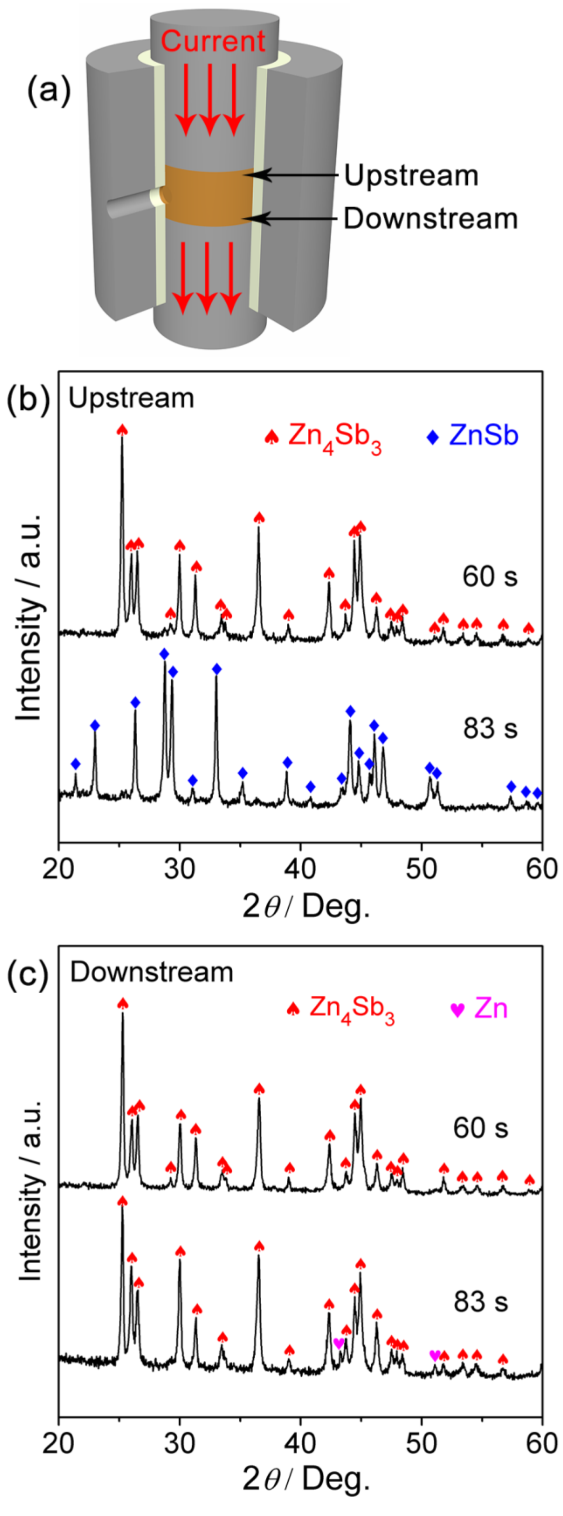

Supplementary Figure 33. Phase composition of the raw material admixture (4Zn + 3Sb) in the upstream and downstream directions of current at different power-on times.

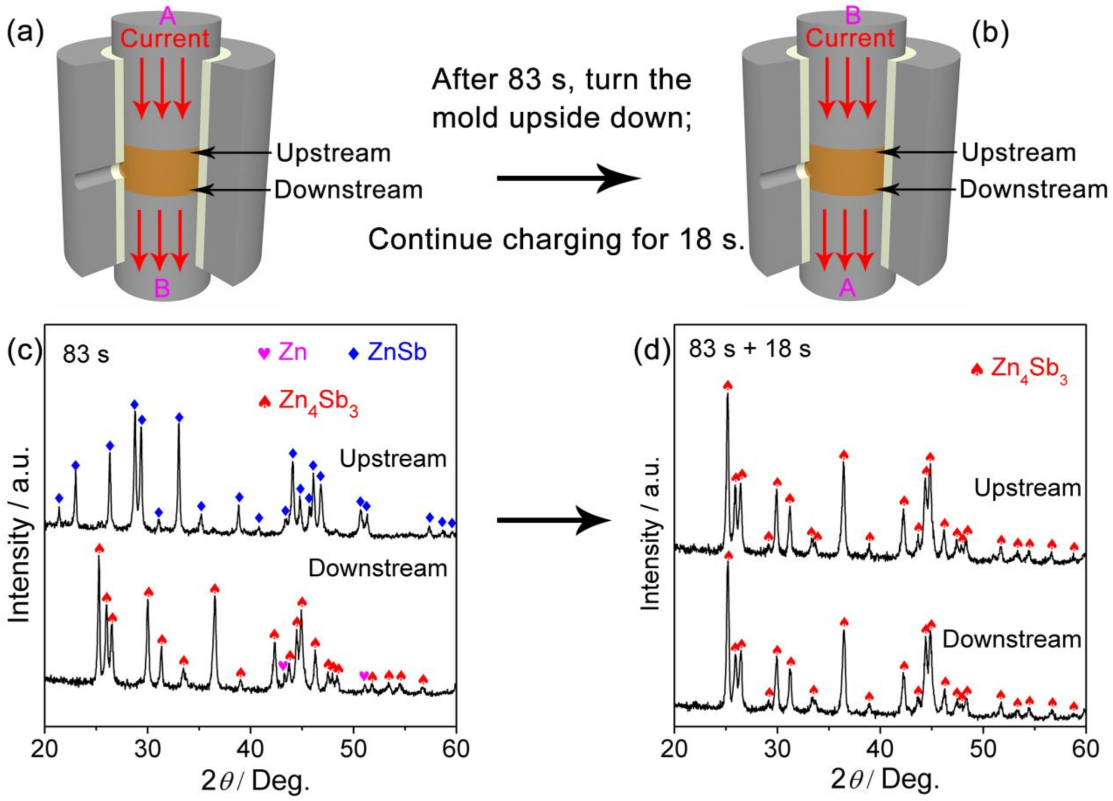

Supplementary Figure 34. Phase composition of the bulk material in the upstream and downstream positions under pulse current. (a) Schematic diagram of the charging process; (b) charging for 83 s; (c) after 83 s, the die with the sample is turned upside down and charging continues for additional 18 s.

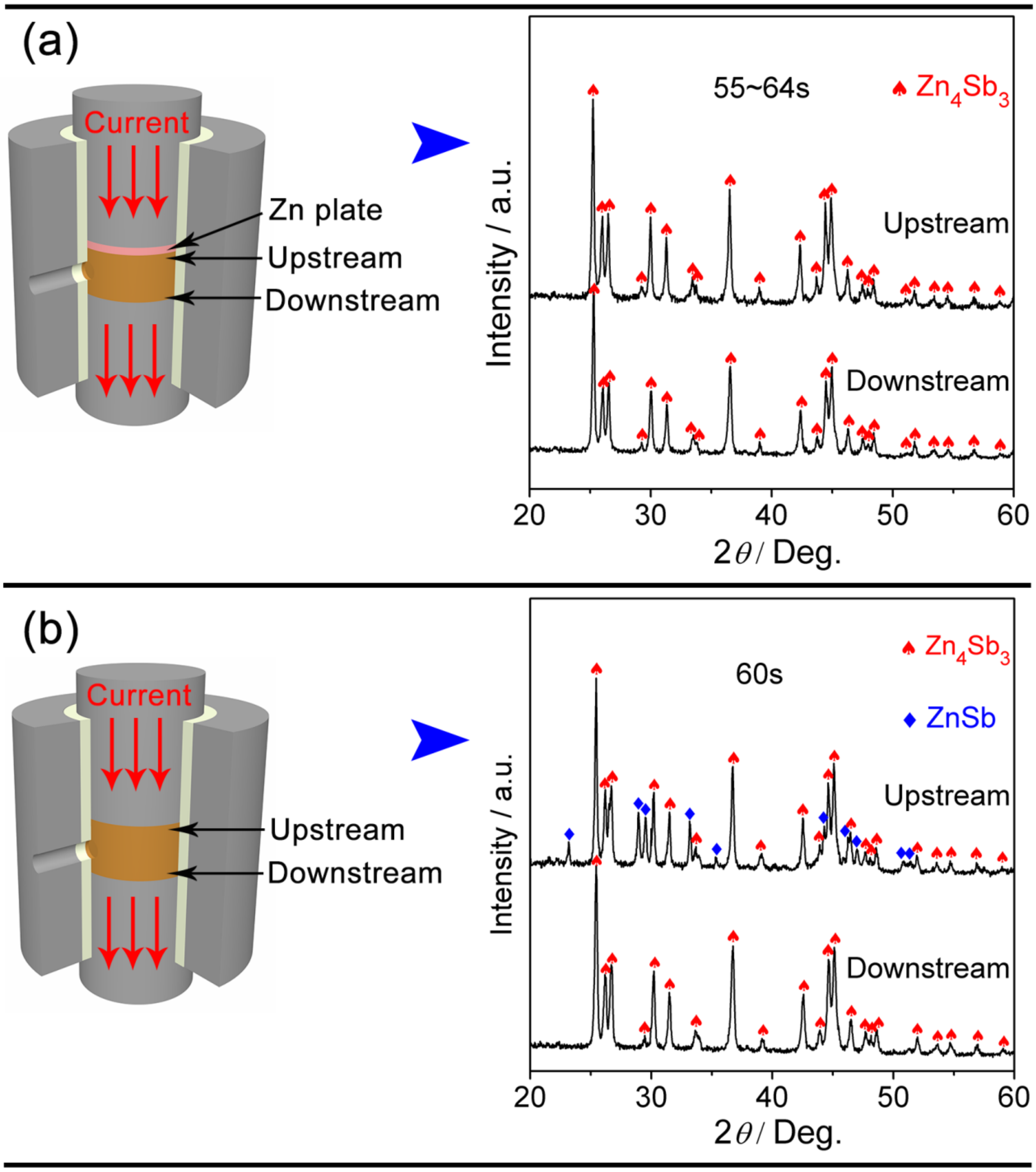

Supplementary Figure 35. Phase composition of the bulk material in the upstream and downstream positions under pulse current. (a) A Zn plate is placed on the top surface of thoroughly mixed stoichiometric powders of "4Zn + 3Sb" inside the graphite mold. (b) Without a Zn plate inside the graphite mold.

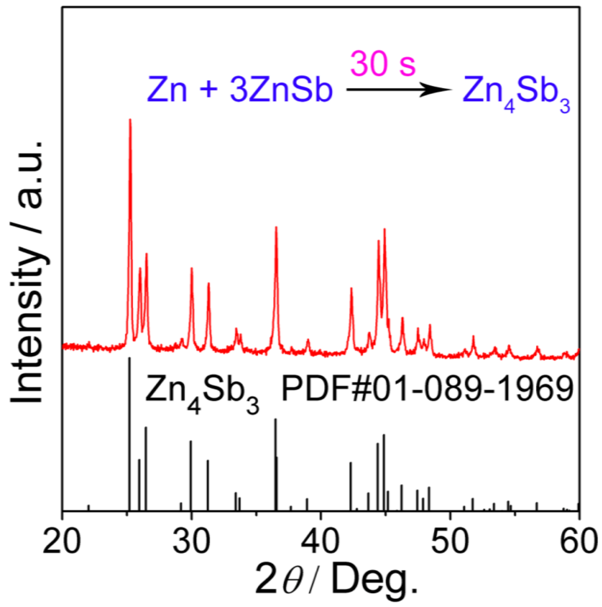

Supplementary Figure 36. Phase composition of the "Zn + 3ZnSb" mixed powder under the pulsed current field applied for 30 s. A single phase Zn_4Sb_3 compound is obtained.

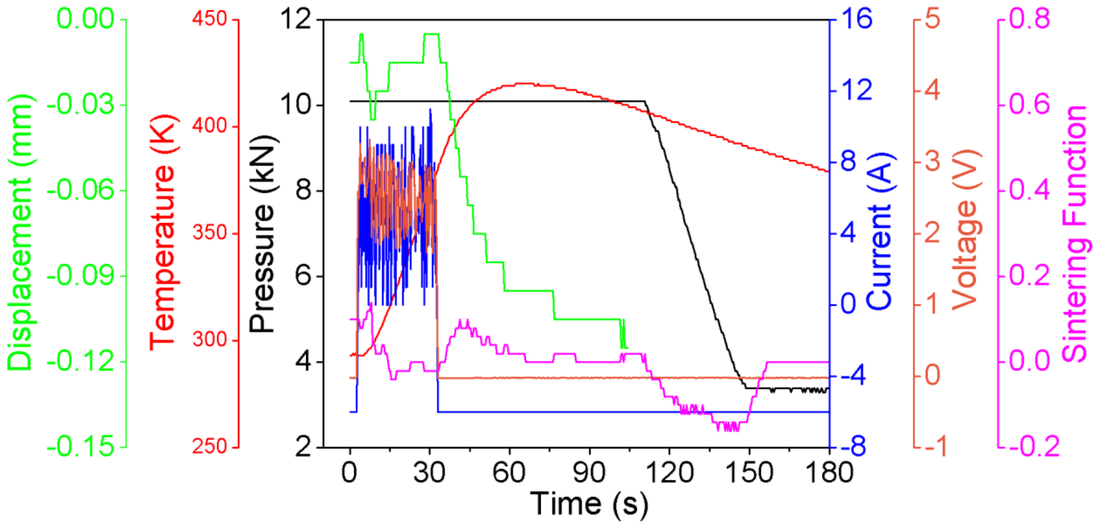

Supplementary Figure 37. Time profile of the reaction parameters of the stoichiometric admixture of " Zn + 3ZnSb " powders under pulse current field.

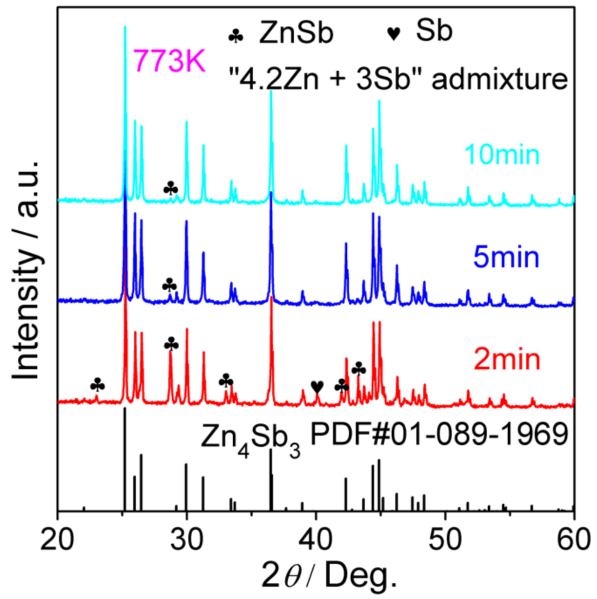

Supplementary Figure 38. XRD patterns of the "4.2Zn + 3Sb" admixture by thermal explosion at 773 K for different times. A nearly single-phase Zn_4Sb_3 compound can be prepared within 10 min.

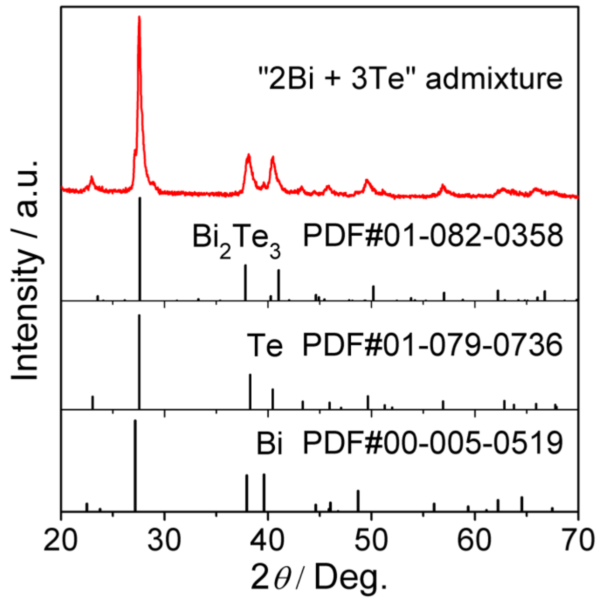

Supplementary Figure 39. XRD pattern of the stoichiometric admixture of "2Bi + 3Te" powders under pulse current field applied for 60 s.

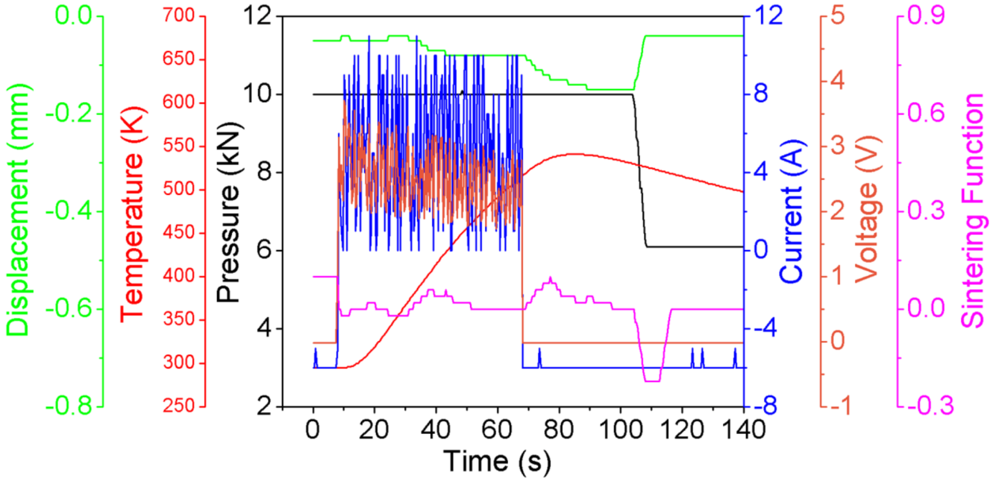

Supplementary Figure 40. Time profile of the reaction parameters of the stoichiometric admixture of "2Bi + 3Te" powders under the pulsed current.

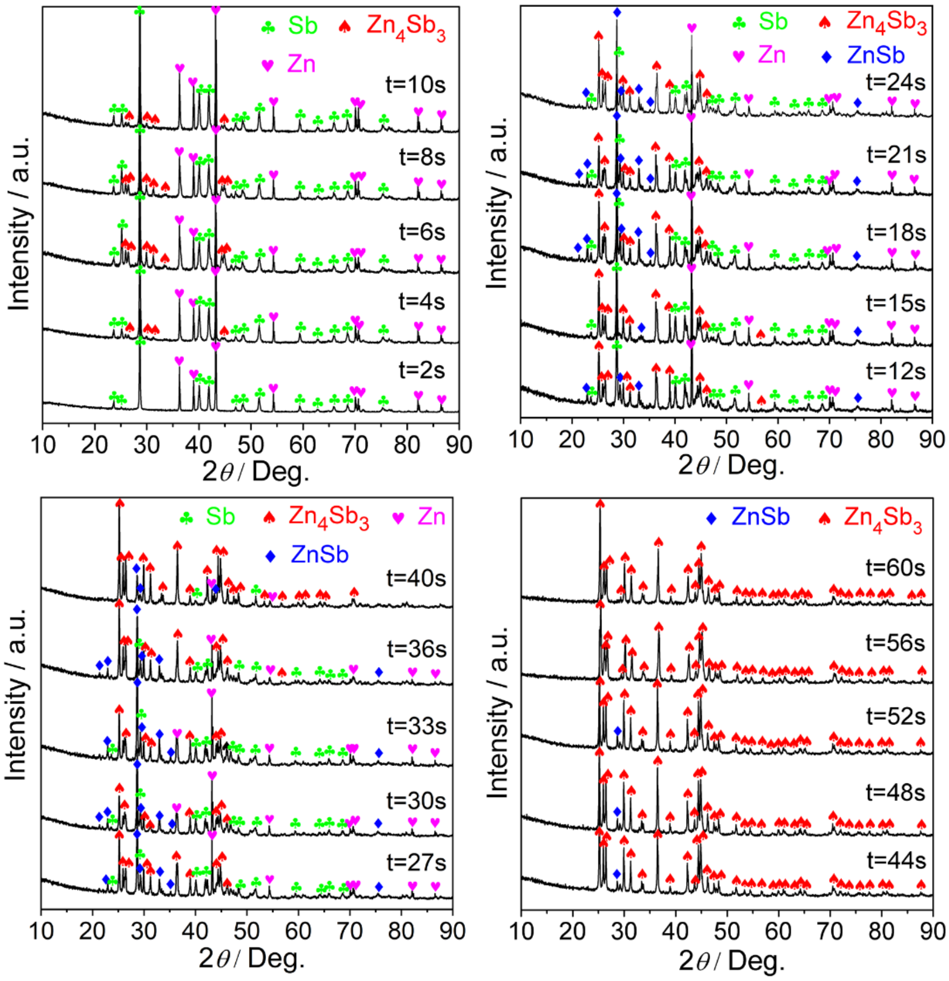

Supplementary Figure 41. Intermediate stages in the phase transformation process of "4Zn + 3Sb" mixed powders under the pulsed current.

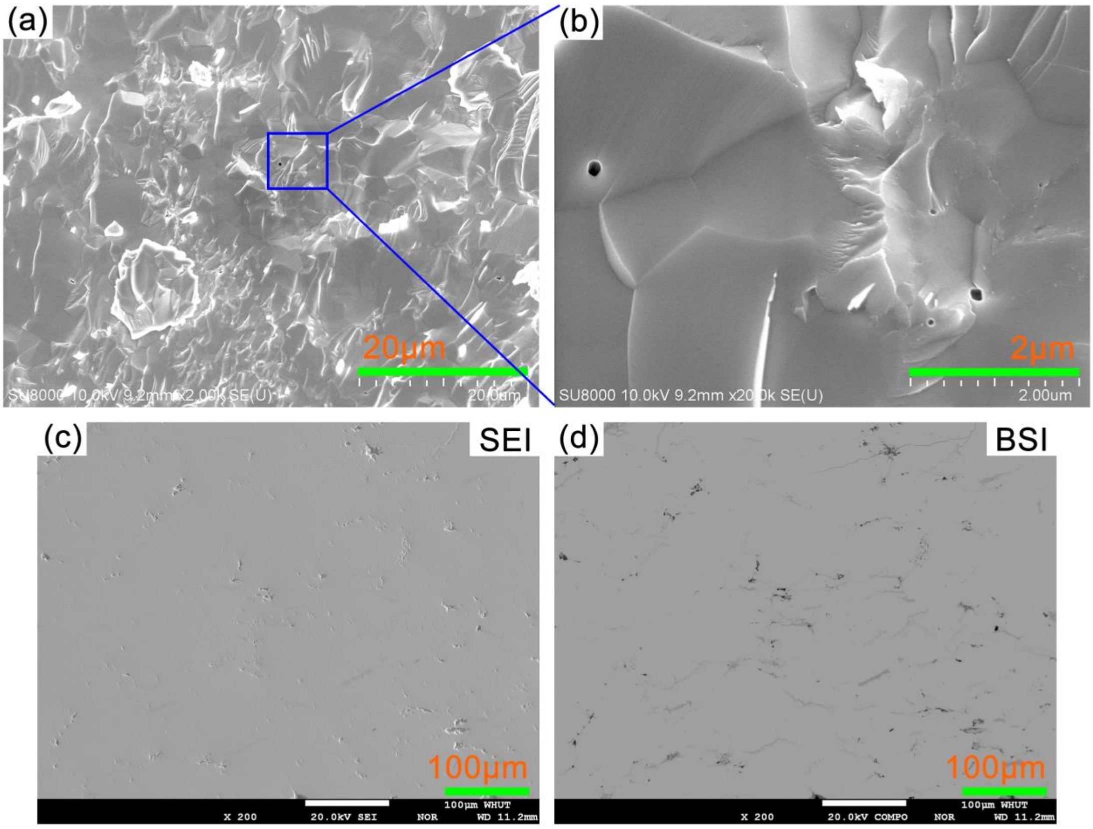

Supplementary Figure 42. (a) Fracture surface morphology of Zn_4Sb_3 prepared by the EFAS process, (b) Enlarged views of the blue-marked regions in (a). (c, d) Secondary electron images and back scattering images of a polished surface of bulk Zn_4Sb_3 prepared by the EFAS process. Microstructures of β-Zn_4Sb_3-based bulk samples synthesized by the EFAS method are similar. All are well compacted with a relative density higher than 98%, and the distribution of elements is uniform

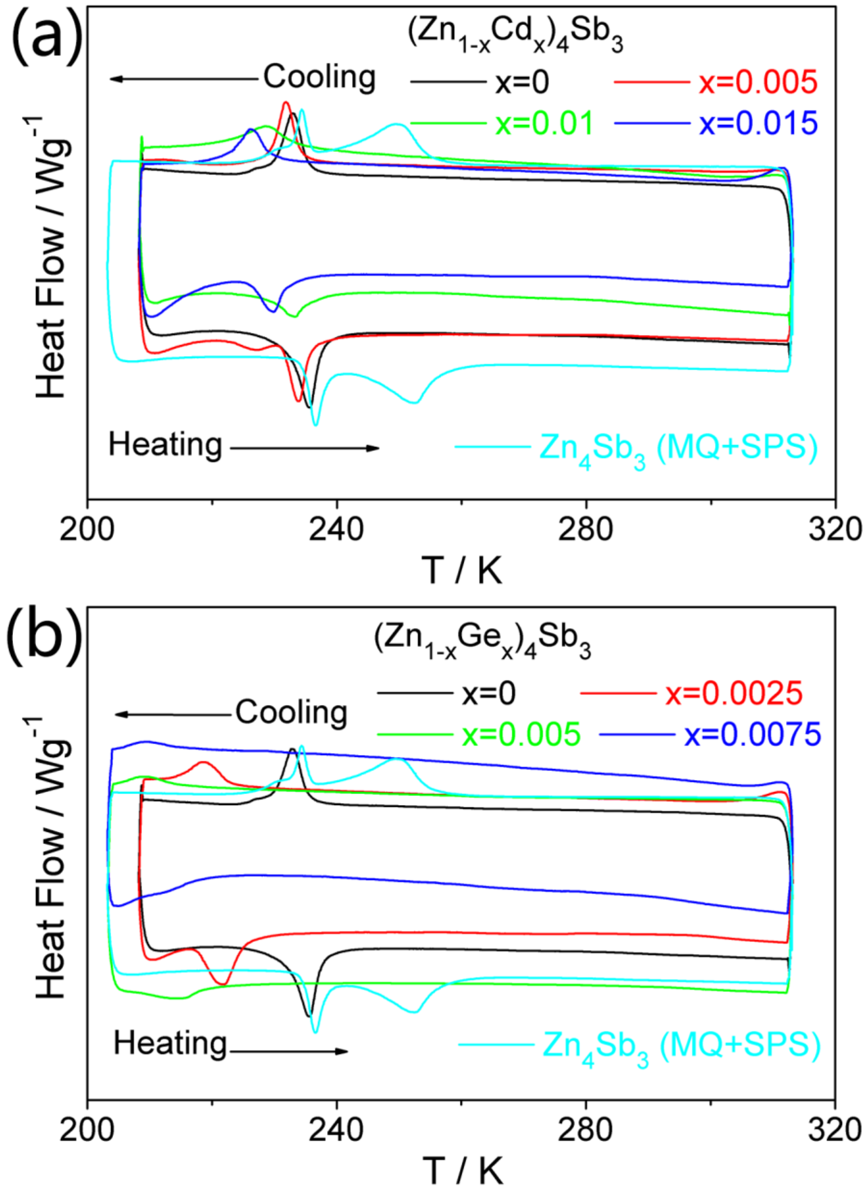

Supplementary Figure 43. Low temperature heat flow curves of Zn_4Sb_3-based compounds: (a) $(Zn_{1-x}Cd_x)_4Sb_3$, (b) $(Zn_{1-x}Ge_x)_4Sb_3$. For comparison, the heat flow curve of the Zn_4Sb_3 (MQ + SPS) sample is also included.

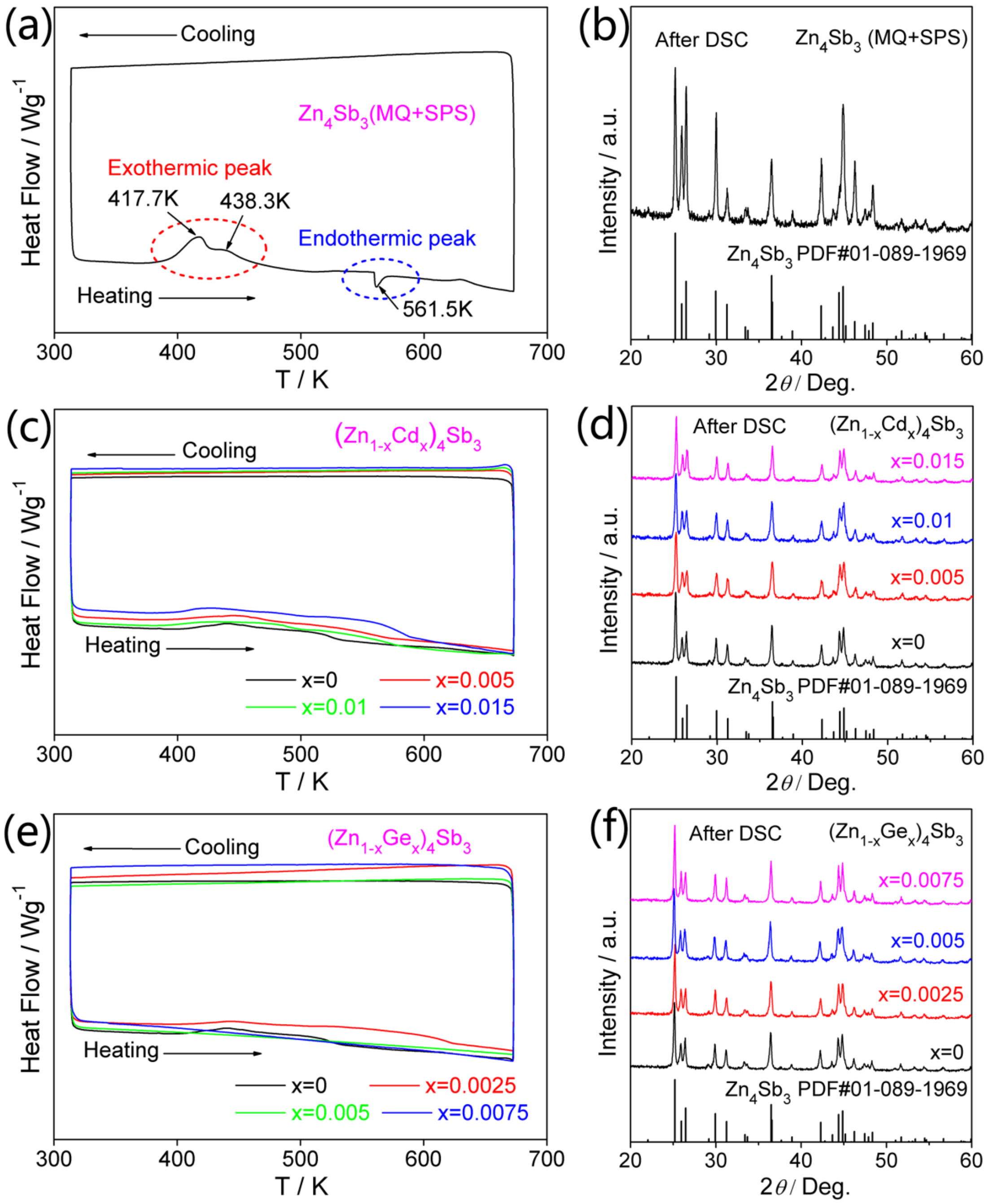

Supplementary Figure 44. High temperature heat flow curves and XRD patterns of Zn_4Sb_3-based compounds after DSC measurement: (a) Zn_4Sb_3 (MQ + SPS), (b) $(Zn_{1-x}Cd_x)_4Sb_3$, (c) $(Zn_{1-x}Ge_x)_4Sb_3$.

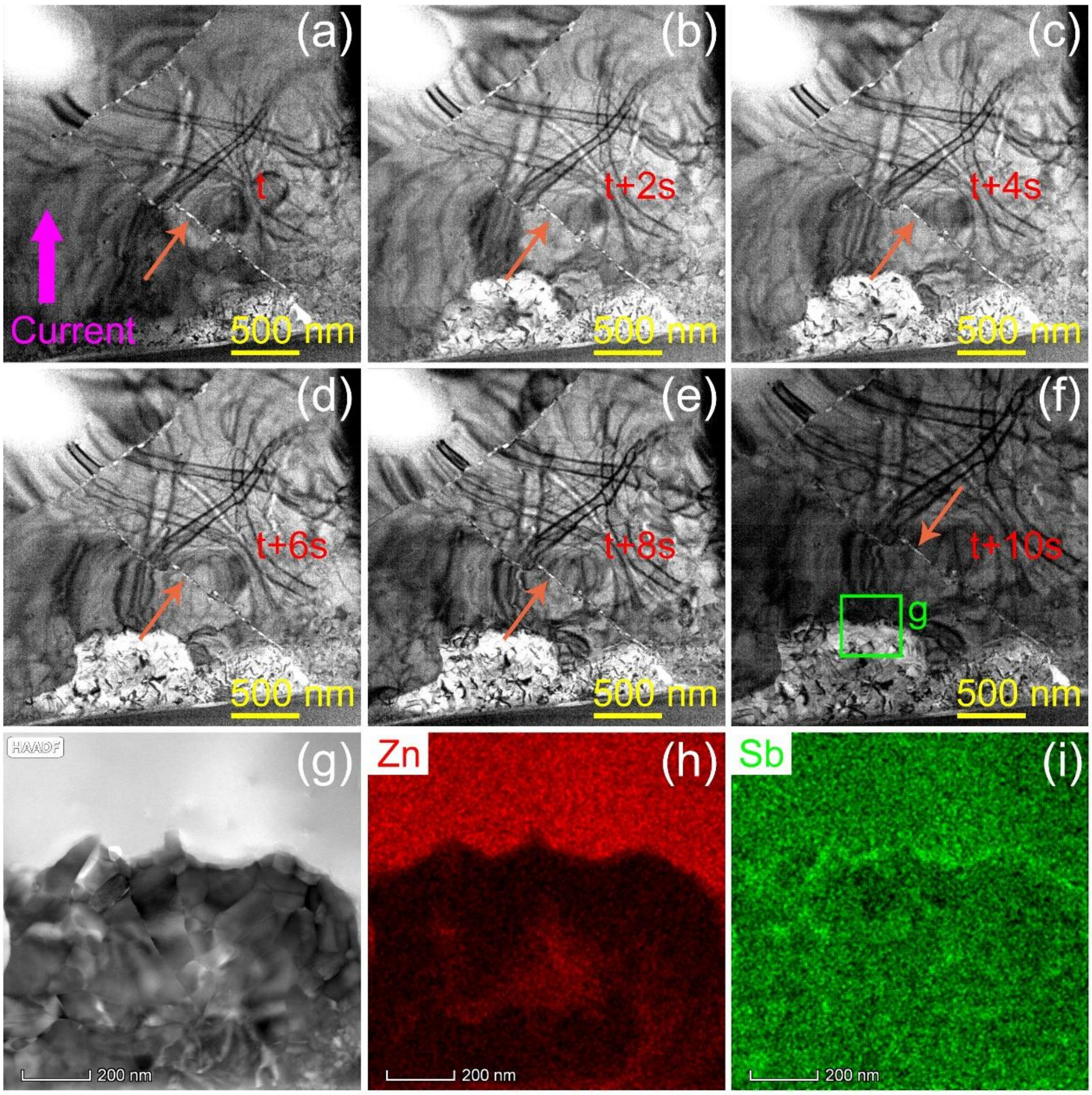

Supplementary Figure 45. In situ TEM images. (a-f) Different stages (in time) during the current flow through β-Zn_4Sb_3 (MQ + SPS). As long as the voltage is applied, the grains will crack and disintegrate continually, and there is no phenomenon of grain boundary widening. (g-i) The HAADF image and the corresponding EDS spectrum of β-Zn_4Sb_3 (MQ + SPS) material after the current flowing through it. It is very obvious that the Zn ions migrate downstream with the current, and the upstream grains crack and fragment due to the lack of a large amount of Zn.

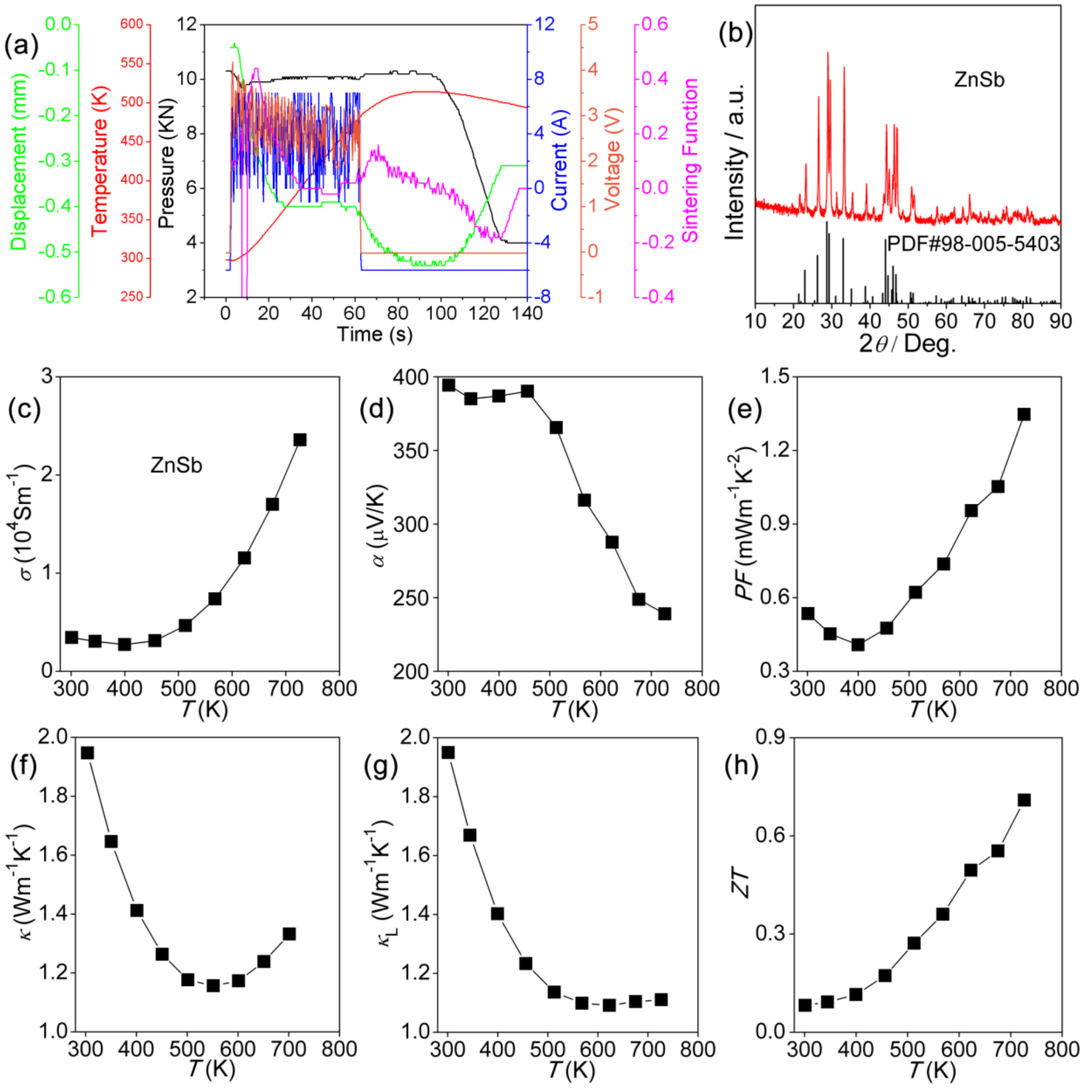

Supplementary Figure 46. ZnSb compound prepared by the EFAS method. (a) Time profile of the reaction parameters, including the loading pressure, temperature of the admixture, displacement of the graphite punch, current, voltage and sintering function (i.e., the derivative of the displacement), (b) XRD pattern of the "Zn + Sb" admixture after charging for 60 s, (c) electrical conductivity, (d) Seebeck coefficient, (e) power factor, (f) total thermal conductivity, (g) lattice thermal conductivity, (h) Fig. of merit *ZT*.

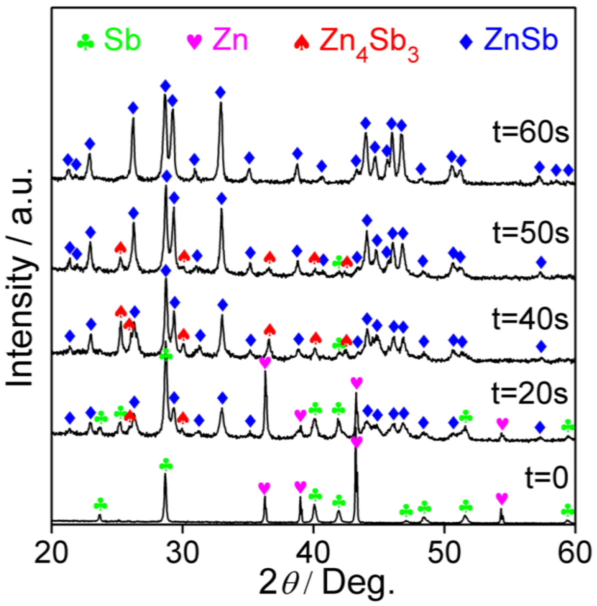

Supplementary Figure 47. Intermediate stages in the phase transformation process of the "Zn + Sb" mixed powder under the pulsed current.

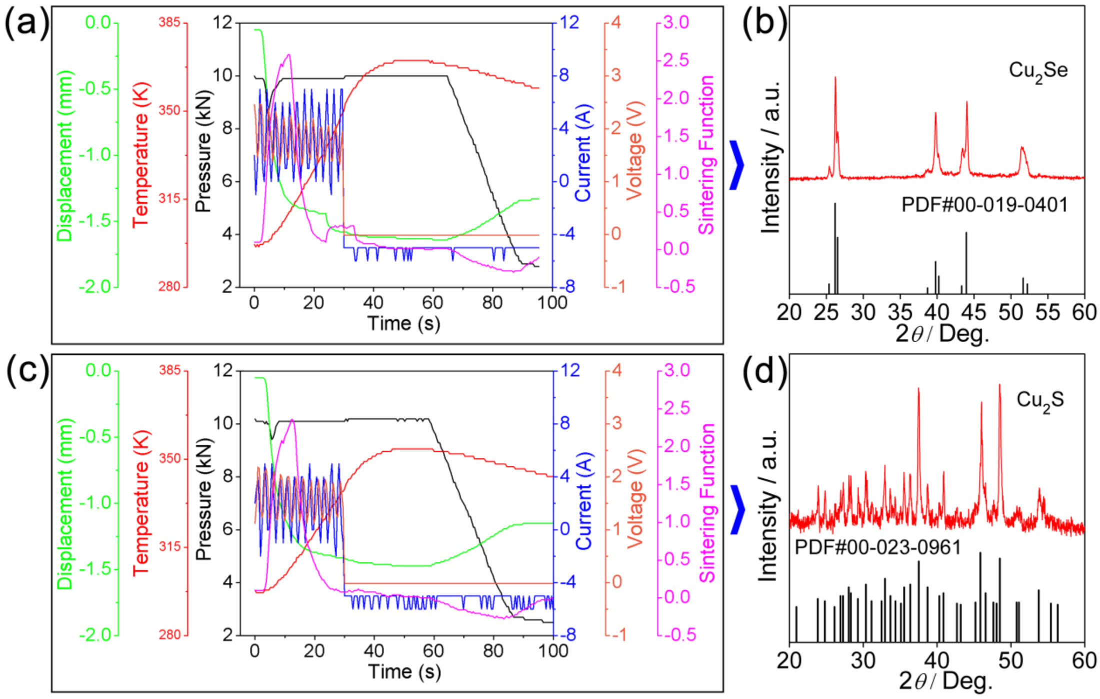

Supplementary Figure 48. Reaction parameters and phase composition of "2Cu + Se" (a, b) and "2Cu + S " (c, d) mixed powder under the pulsed current.

Supplementary References:

1. Wang, S. Y., Li, H., Qi, D. K., Xie, W. J. & Tang, X. F. Enhancement of the thermoelectric performance of β-Zn_4Sb_3 by in situ nanostructures and minute Cd-doping. *Acta Mater.* **59**, 4805-4817 (2011).

2 Wang, S. Y., Tan, X. J., Tan, G. J., She, X. Y., Liu, W., Li, H., Liu, H. J. & Tang, X. F. The realization of a high thermoelectric Fig. of merit in Ge-substituted β-Zn_4Sb_3 through band structure modification. *J. Mater. Chem.* **22**, 13977-13985 (2012).

3 Kamaya, N., Homma, K. J., Yamakawa, Y., Hirayama, M., Kanno, R., Yonemura, M., Kamiyama, T., Kato, Y., Hama, S., Kawamoto, K. & Mitsui, A A lithium superionic conductor. *Nat. Mater.* **10**, 682-686 (2011).

4 Murugan, R., Thangadurai, V. & Weppner, W. Fast lithium ion conduction in garnet−type $Li_7La_3Zr_2O_{12}$. *Angew. Chem. Int. Edit.* **46**, 7778-7781 (2007).

5 Seino, Y., Ota, T., Takada, K., Hayashi, A. & Tatsumisago, M. A sulphide lithium super ion conductor is superior to liquid ion conductors for use in rechargeable batteries. *Energ. Environ. Sci.* **7**, 627-631 (2014).

6 Zhang, Z., Ramos, E., Lalere, F., Assoud, A., Kaup, K., Hartman, P. & Nazar, L. F. $Na_{11}Sn_2PS_{12}$: a new solid state sodium superionic conductor. *Energ. Environ. Sci.* **11**, 87-93 (2018).

7 Yin, H., Blichfeld, A. B., Christensen, M. & Iversen, B. B. Fast direct synthesis and compaction of homogenous phase-pure thermoelectric Zn_4Sb_3. *ACS Appl. Mater. Interfaces* **6**, 10542-10548 (2014).

8 Lin, J. P., Ma, L. Z., Zheng, Z. H., Chen, Y. L., Cui, Z. C., Wang, J. P. & Qiao, G. J. Thermoelectric Properties of Zn_4Sb_3 Composites with Incomplete Reaction. *J. Electron. Mater.* **48**, 1159-1163 (2018).

9 Su, X. L., Fu, F., Yan, Y. G., Zheng, G., Liang, T., Zhang, Q., Cheng, X., Yang, D. W., Chi, H., Tang, X. F., Zhang, Q. J. & Uher, C. Self-propagating high-temperature synthesis for compound thermoelectrics and new criterion for combustion processing. *Nat. Commun.* **5**, 4908-4914 (2014).

10 Zheng, G., Su, X. L., Liang, T., Lu, Q. B., Yan, Y. G., Uher, C. & Tang, X. F. High thermoelectric performance of mechanically robust n-type $Bi_2Te_{3-x}Se_x$ prepared by combustion synthesis. *J. Mater. Chem. A* **3**, 6603-6613 (2015).

11 Lin, J. P., Li, X. D., Qiao, G. J., Wang, Z., Carrete, J., Ren, Y., Ma, L. Z., Fei, Y. J., Yang, B. F., Lei, L. & Li, J. Unexpected high-temperature stability of β-Zn4Sb3 opens the door to enhanced thermoelectric performance. *J. Am. Chem. Soc.* 136, 1497-1504 (2014).
