# Supplementary material for: Fast ion transport for synthesis and stabilization of β-Zn4Sb3
Source: Nat Commun. 2021 Oct 19;12:6077. doi: 10.1038/s41467-021-26265-0 (PMC8526605; doi:10.1038/s41467-021-26265-0)
Supplement: Supplementary file 2 — Description of Additional Supplementary Files [file 41467_2021_26265_MOESM2_ESM.docx]

**Supplementary Movie 1**:

In situ high resolution transmission electron microscopy of the EFAS Zn_4_Sb_3_ sample

**Supplementary Movie 2**:

In situ high resolution transmission electron microscopy of the MQ + SPS Zn_4_Sb_3_ sample
